# Latent Profiles of Attitudes Toward Artificial Intelligence Applications Among Traditional Chinese Medicine Nurses and Associated Factors: A Multicenter Cross‐Sectional Study

**DOI:** 10.1155/jonm/6814652

**Published:** 2026-11-04

**Authors:** Shuang Lu, Xian Liu, Longxian Liu, Xinrong Tan, Guo Yu, Wenxia Hu, Meigui Peng, Ziyi Yuan, Shaoyang Cui, Meng Wu, Zining Guo

**Affiliations:** ^1^ Department of Nursing Shenzhen Hospital of Guangzhou University of Chinese Medicine (Futian) Shenzhen Guangdong China; ^2^ The Sixth Clinical College of Guangzhou University of Chinese Medicine Guangzhou Guangdong China; ^3^ Maternal and Child Health Hospital of Futian District Shenzhen Guangdong China

**Keywords:** artificial intelligence, associated factors, attitude, cross-sectional study, latent profile analysis, nurses, traditional Chinese medicine nursing

## Abstract

**Background:**

Artificial intelligence (AI) technologies are increasingly being integrated into clinical nursing practice. However, nurses with different professional backgrounds may differ in their attitudes toward AI applications. Traditional Chinese medicine (TCM) nurses, whose practice integrates traditional Chinese nursing techniques, may have distinct perceptions and levels of acceptance of AI compared with general clinical nurses.

**Methods:**

A multicenter cross‐sectional survey was conducted from 10 January to 7 April 2026 in three hospitals and 30 community health service centers in South China. A total of 590 TCM nurses were included. Data were collected using a self‐designed general information questionnaire and standardized scales. Latent profile analysis was performed using the 15‐item scores of the Attitude Scale Toward the Use of Artificial Intelligence Technologies in Nursing as observed indicators. Associations with profile membership were examined using R3STEP to account for classification uncertainty, with simultaneous adjustment for 13 covariates.

**Results:**

The mean total score for attitudes toward AI applications among TCM nurses was 48.13 ± 13.49. Three latent profiles were identified: the low AI acceptance profile (25.14%), the moderate AI acceptance profile (39.68%), and the high AI acceptance profile (35.18%). R3STEP analyses identified profile‐specific associations of TCM specialist nursing experience, education, professional title, TCM nursing professional level, intelligent‐device use, self‐rated familiarity with AI, AI‐related training, and technology anxiety with profile membership.

**Conclusions:**

TCM nurses demonstrated three latent profiles of attitudes toward AI applications: low, moderate, and high AI acceptance. Profile membership was associated with several occupational and technology‐related characteristics, with patterns varying across profile comparisons. These findings may inform AI training and implementation that take differences in nurses’ acceptance into account.

## 1. Introduction

With the continued advancement of artificial intelligence (AI) in health care, its application in clinical nursing has progressed from conceptual exploration toward practical implementation [1]. In recent years, technologies such as electronic health record management systems, nursing robots, intelligent clinical decision support systems, and telehealth platforms have increasingly been integrated into nursing practice and may improve the quality and efficiency of nursing care [2, 3]. As AI becomes more deeply integrated into clinical practice, nurses’ attitudes toward AI may be associated with their willingness to adopt and use these technologies [4]. However, existing research has focused primarily on nurses in general clinical settings, nursing students, and other healthcare professionals [5–7]. These findings may not fully reflect the perspectives of traditional Chinese medicine (TCM) nurses, whose practice is shaped by distinctive professional and cultural traditions.

TCM nursing is guided by TCM theory, rooted in traditional Chinese culture, and characterized by an emphasis on holistic care, syndrome differentiation‐based nursing, regulation of yin and yang, individualized care, and humanistic care [8–10]. In addition to considering disease diagnoses and physiological indicators, TCM nurses comprehensively assess patients’ constitutions, symptoms, emotional states, lifestyles, environmental factors, and information derived from the four diagnostic methods. Based on these assessments, they provide individualized care using TCM nursing techniques such as moxibustion, auricular acupressure, acupoint application, and Tuina [10–12]. Accordingly, AI applications in TCM nursing need to be compatible with the professional characteristics of holistic assessment, syndrome differentiation–based nursing, individualized interventions, and humanistic care [13–15]. Uncertainty regarding this compatibility, together with concerns about professional accountability, data security, and quality control, may make TCM nurses more cautious about adopting AI [16, 17].

The technology acceptance model (TAM), proposed by Davis in 1989, identifies perceived usefulness and perceived ease of use as two core constructs related to technology acceptance [18]. In the context of TCM nursing, perceived usefulness concerns whether AI can assist in integrating information from the four diagnostic methods, conducting syndrome assessments, and optimizing syndrome differentiation–based nursing plans. Perceived ease of use concerns whether AI technologies are easy to learn and operate and can be integrated into existing nursing workflows. TCM nurses’ combined assessments of the clinical value and ease of use of AI may therefore shape their attitudes toward its application.

Rather than summarizing attitudes using a single total score, latent profile analysis uses individuals’ response patterns across multiple items to identify latent subgroups with similar characteristics [19]. This method has previously been used to identify distinct profiles of attitudes toward AI among nurses in general clinical settings [20]. However, evidence specific to TCM nursing remains scarce. The limited existing studies have focused primarily on TCM physicians and other traditional medicine–related groups. Their findings suggest that although these groups recognize the potential applications of AI, they also have concerns about technological reliability and compatibility with traditional medicine practice [21, 22]. None of these studies specifically examined TCM nurses, and they primarily described attitudes at the aggregate level without investigating distinct individual response patterns across multiple attitude items. Because TCM nurses may evaluate AI from several perspectives—including its clinical utility, compatibility with TCM nursing practice, and potential risks—a person‐centered latent profile approach is particularly well suited to the purpose of this study.

Accordingly, this study used latent profile analysis to identify distinct profiles of attitudes toward AI applications among TCM nurses. We further examined the associations of demographic characteristics, TCM nursing–related experience, AI‐related experience, technology anxiety, general self‐efficacy, and perceived organizational support with profile membership. The findings may help clarify individual heterogeneity in TCM nurses’ attitudes toward AI applications and provide preliminary evidence to inform AI implementation, targeted training, and nursing management strategies in TCM nursing.

## 2. Methods

### 2.1. Study Design

This was a multicenter cross‐sectional survey of TCM nurses. Guided by the TAM, a questionnaire survey was used to collect information on demographic characteristics, AI‐related experience, technology anxiety, perceived organizational support, general self‐efficacy, and attitudes toward the use of AI technologies in nursing. Item scores from the Attitude Scale Toward the Use of Artificial Intelligence Technologies in Nursing (ASUAITIN) were used as observed indicators to identify latent profiles of attitudes toward AI applications among TCM nurses and to examine associated factors. The study was reported in accordance with the Strengthening the Reporting of Observational Studies in Epidemiology (STROBE) statement for cross‐sectional studies to ensure complete, standardized, and transparent reporting of the study process and findings [23, 24]. The completed STROBE checklist is provided in Supporting File 9.

### 2.2. Participants

This study was conducted from January to April 2026 in multiple TCM‐related medical institutions in South China, including Shenzhen Hospital of Guangzhou University of Chinese Medicine (Futian) and its 30 affiliated community health service centers, Maternal and Child Health Hospital of Futian District, and Guangdong Second Traditional Chinese Medicine Hospital. A list of the 30 participating community health service centers is provided in Supporting File 4. Eligible TCM nurses were recruited using multicenter convenience sampling, and data were collected through a questionnaire survey.

The inclusion criteria were as follows: (1) holding a valid nurse practice certificate and working as a frontline clinical nurse; (2) being a certified TCM specialist nurse who had completed training and assessment at a TCM nursing specialist nurse training base, or having received systematic education in TCM nursing or integrated traditional Chinese and Western medicine nursing; (3) currently working in a TCM hospital, TCM department, integrated traditional Chinese and Western medicine department, or other clinical department providing TCM nursing services; (4) having engaged in TCM specialist nursing for at least 6 months and participating in TCM nursing assessment, syndrome‐based nursing, or appropriate TCM nursing techniques in daily work; and (5) providing informed consent and voluntarily participating in the study.

The exclusion criteria were as follows: (1) incomplete questionnaire responses or missing key variables; (2) an obviously short response time or clear patterned responses; (3) duplicate questionnaire submissions by the same respondent, in which case only the first valid questionnaire was retained; and (4) obvious logical inconsistencies between demographic information and questionnaire responses.

### 2.3. Sample Size

The sample size was estimated using the single‐proportion formula: $n_{0}=Z_{12-\alpha /}^{2}\times Ρ\left(1-Ρ\right)/d^{2}$ [25]. With (α = 0.05), (*Ζ*_1−*α*/2_ = 1.96), (*p* = 0.50), and (*d* = 0.05), the initial sample size was calculated as (*n*_0_ = 384.16). Allowing for 20% invalid questionnaires, the adjusted sample size was (*n* =  *n*_0_/(1–0.20) = 480.20), which was rounded up to 481 participants.

There is no universal minimum sample‐size criterion for LPA; sample‐size requirements depend on the number of indicators, profile separation, profile proportions, and model parameterization [26–28]. A sample size of approximately (*N* = 500) may serve as a practical heuristic [26]. The final analytic sample comprised 590 participants. In the three‐profile model, the smallest profile accounted for 25.14% of the sample, entropy was 0.939, and the average posterior probabilities for the three profiles were 0.967, 0.972, and 0.977, respectively. The best log‐likelihood value was successfully replicated, and the sensitivity analyses yielded stable results. Together, these findings support the practical adequacy of the current sample for estimating the retained three‐profile solution.

### 2.4. Measures

#### 2.4.1. Sociodemographic and Occupational Characteristics

A self‐designed general information questionnaire was used to collect participants’ demographic characteristics, occupational information, and AI‐related experience. The questionnaire included age, gender, educational level, professional title, years of TCM specialist nursing experience, systematic TCM nursing training, TCM nursing professional level, frequency of using intelligent devices in TCM nursing, self‐rated familiarity with AI, and history of AI training. Details are provided in Supporting File 1.

#### 2.4.2. Attitude Scale Toward the Use of Artificial Intelligence Technologies in Nursing

Attitudes toward AI applications in nursing were assessed using the 15‐item Chinese version of ASUAITIN (ASUAITIN‐C). The original scale was developed by Yılmaz et al. [29]. Wang et al. [30] translated and culturally adapted the scale through independent forward translation, reconciliation, independent back‐translation, comparison with the source version, review by the original developer, expert consultation, and pilot testing in 30 nurses. Items 3, 10, 11, and 13 were linguistically adapted for the Chinese nursing context. The scale comprises six negatively worded items (items 1–6) and nine positively worded items (items 7–15), each rated from 1 = Strongly Disagree to 5 = Strongly Agree. Items 1–6 were reverse‐scored as 6 minus the response, yielding a possible total score of 15–75; higher scores indicate a more favorable attitude toward AI applications in nursing. The 15 scored item responses were subsequently used as continuous observed indicators in the LPA. The complete administered item set, domain mapping, response format, scoring key, and sample‐specific psychometric results are provided in Supporting File 5.

#### 2.4.3. Perceived Organizational Support Scale

Perceived organizational support was measured using the Perceived Organizational Support Scale revised by Zuo et al. [31]. The scale assesses the extent to which participants perceive organizational recognition, concern, and resource support in the workplace. It includes two dimensions, emotional support and instrumental support, with 13 items in total. Each item is rated on a 5‐point Likert scale, ranging from 1 = Strongly Disagree to 5 = Strongly Agree. The total score ranges from 13 to 65, with a higher score indicating a higher level of perceived organizational support. Previous research reported good internal consistency for this scale, with a Cronbach’s *α* coefficient of 0.920. In the present sample, Cronbach’s *α* for the 13‐item scale was 0.950.

#### 2.4.4. Technology Anxiety Scale

Technology anxiety was assessed using 13 Chinese items derived from the Technophobia Scale originally developed by Khasawneh [32] and translated and culturally adapted by Sun et al. [33] through translation, back‐translation, expert review, and cross‐cultural adaptation. The instrument comprises technology‐related tension (items 1–5), fear of technology (items 6–10), and privacy and security concerns (items 11–13). In the present survey, the items were rated on a 5‐point Likert scale ranging from 1 (Strongly Disagree) to 5 (Strongly Agree). No item was reverse‐scored. Item scores were summed to yield a possible total score of 13–65, with higher scores indicating greater technology anxiety. The total score was entered as a continuous covariate in the R3STEP analysis. The complete administered item set, dimension mapping, response format, scoring key, and sample‐specific psychometric results are provided in Supporting File 5.

#### 2.4.5. General Self‐Efficacy Scale

General self‐efficacy was measured using the General Self‐Efficacy Scale developed by Schwarzer et al. [34] and revised in Chinese by Wang et al. [35]. The scale assesses individuals’ confidence and self‐regulatory capacity when facing difficult tasks and work‐related challenges. It contains 10 items, each rated on a 4‐point Likert scale, ranging from 1 = Completely Incorrect to 4 = Completely Correct. The total score ranges from 10 to 40, with a higher score indicating stronger general self‐efficacy. Previous research reported acceptable internal consistency for the scale, with a Cronbach’s *α* coefficient of 0.800. In the present sample, the 10‐item scale had a Cronbach’s *α* of 0.927.

### 2.5. Data Collection and Quality Control

Data were collected online using the Wenjuanxing platform (https://www.wjx.cn/login.aspx) from January 10, 2026, to April 7, 2026. After permission was obtained from the relevant administrative departments of each participating center, the researchers distributed the questionnaire link or QR code to eligible clinical nurses through departmental head nurses. The first page of the questionnaire provided information on the study purpose, study content, instructions for completion, anonymity, and data confidentiality. All participants completed the questionnaire voluntarily after providing informed consent. To improve data quality, respondents accessed the survey through WeChat, with only one submission permitted per WeChat account, and all items were set as mandatory. Details of the questionnaire‐screening criteria are provided in the Quality Control Measures subsection. To reduce potential selection bias, the survey was conducted simultaneously in multiple hospitals and their affiliated community health service institutions, with efforts made to include TCM nurses from different departments, professional titles, professional levels, and working years. To reduce information bias and social desirability bias associated with self‐reported questionnaires, the survey was anonymous, and the questionnaire instructions emphasized that there were no right or wrong answers and that participants should respond independently according to their actual situation. To reduce common method bias, different scale modules were used to measure the study variables, leading statements were avoided, and questionnaires with poor response quality or logical abnormalities were excluded during data cleaning.

### 2.6. Statistical Analysis

Statistical analyses were performed using SPSS 27.0, Mplus 8.3, and R 4.2.3. All statistical tests were two‐sided, with a significance level of *α* = 0.05. A *p* value of < 0.05 was considered statistically significant. Normality of continuous variables was assessed using the Shapiro–Wilk test, supplemented by visual inspection of histograms and normal Q–Q plots. Categorical variables were summarized as frequencies and percentages and compared using Pearson’s chi‐square test or Fisher’s exact test, as appropriate. As the continuous variables did not meet the assumption of normality, they were summarized as medians and interquartile ranges (IQRs) and compared across latent profiles using the Kruskal–Wallis test.

Internal consistency was evaluated using Cronbach’s alpha. For post hoc internal validation, the sample was randomly divided into derivation and validation subsets (*n* = 295 each; seed = 20260826) [36]. A one‐factor model was estimated in the derivation subset using robust maximum likelihood (MLR). The estimated factor loadings, item residual variances, and item intercepts were then fixed in the validation subset, while the latent mean and variance were freely estimated. Analyses were conducted in R Version 4.2.3 using lavaan Version 0.6–19 [37]. Model fit was evaluated using the robust *χ* ^2^ statistic, CFI, TLI, RMSEA with its 90% confidence interval, and SRMR [38]. Composite reliability (CR) and average variance extracted (AVE) were calculated from the derivation‐subset estimates [39]. The 15 scored items from the Attitude Scale toward the Use of Artificial Intelligence Technologies in Nursing (ASUAITIN‐C) were entered individually as continuous observed indicators in the LPA conducted using Mplus 8.3. Item‐level indicators were selected to retain multivariate response information across the scale that would otherwise be collapsed into a single value by use of the total score, thereby permitting potential heterogeneity in item‐level response patterns to be evaluated empirically [19]. The primary LPA models were specified under the local independence assumption: Class‐specific item means were freely estimated, the residual variance of each item was constrained to be equal across profiles, and all within‐profile residual covariances were fixed at zero. Models with one through five profiles were estimated sequentially. The optimal model was determined by comparing fit indices across models with different numbers of profiles, including entropy, the Lo–Mendell–Rubin (LMR) likelihood ratio test, the bootstrap likelihood ratio test (BLRT), the Akaike information criterion (AIC), the Bayesian information criterion (BIC), and the sample‐size‐adjusted Bayesian information criterion (aBIC) [40–42]. Lower AIC, BIC, and aBIC values indicated better model fit. Significant LMR test and BLRT results suggested that the current model provided a better fit than the model with one fewer profile. Entropy was used to assess classification accuracy, with values closer to 1 indicating better profile separation. The final model was selected based on fit indices, classification accuracy, profile size, and theoretical interpretability. To examine whether the retained solution was susceptible to prominent violations of local independence, we conducted a targeted sensitivity analysis. The residual covariances for Q1–Q4, Q2–Q6, and Q7–Q15 were freely estimated and constrained to be equal across profiles; all remaining within‐profile residual covariances were fixed at zero. After the optimal model was determined, the profiles were named according to the mean score patterns across the 15 items. Line plots of item mean scores were generated using R 4.2.3 to show the response patterns of different latent profiles. Differences in demographic characteristics, AI‐related experience, general self‐efficacy, technology anxiety, and perceived organizational support across latent profiles were then examined. Associations with latent profile membership were examined using the bias‐adjusted R3STEP procedure to account for classification uncertainty. All 13 a priori covariates, selected on the basis of the TAM, prior nursing AI research, and the TCM nursing context, were entered simultaneously; univariate comparisons were descriptive only. The analytic coding and reference categories are provided in Supporting File 2, and details of the recategorization of categorical covariates are provided in Supporting File 6. The low AI acceptance profile (Profile 1) was selected as the reference category because it showed the lowest overall level of AI acceptance. Adjusted odds ratios were interpreted for Profile 2 versus Profile 1 and Profile 3 versus Profile 1. Self‐reported intelligent‐device use in TCM nursing was analyzed as a four‐category variable, with “1–2 times per week” as the reference category. Separate comparisons were made for “3–4 times per week,” “5–6 times per week,” and “regularly used as routine practice.” The routine‐practice category was not assigned a numerical weekly frequency, and no ordered frequency gradient across all four categories was assumed. As sensitivity analyses, the LPA was re‐estimated using two‐domain mean scores and CFA‐derived factor scores, and R3STEP was repeated using a theory‐informed core set of nine covariates, with details provided in Supporting File 8.

### 2.7. Ethical Considerations

The study was approved by the Ethics Committee of Shenzhen Hospital of Guangzhou University of Chinese Medicine (Futian) on January 9, 2026 (Approval No. GZYLL(KY)‐2026‐003), before participant recruitment and data collection commenced. The ethics approval document is provided in Supporting File 3. All study procedures were conducted in accordance with the Declaration of Helsinki. Before completing the online questionnaire, participants were informed of the study purpose and procedures, voluntary participation, anonymity, and data confidentiality, and provided electronic informed consent. Participants could discontinue the questionnaire at any time before submission. Questionnaire responses were collected anonymously, and no directly identifying information was included in the research dataset.

### 2.8. Quality Control Measures

All questionnaire items were mandatory; therefore, no item‐level missing data were present in the final analytic sample, and neither imputation nor full‐information maximum likelihood was required. Respondents accessed the survey through WeChat, with only one submission permitted per WeChat account. Submitted questionnaires were screened for completion time, logical consistency, and response patterns. Questionnaires were excluded if they met any of the following criteria: (1) a completion time of less than 100 s; (2) logically incompatible responses regarding age and years of work in TCM specialty nursing; or (3) patterned responding. Patterned responding was defined as selecting the same response option throughout a consecutive set of items containing both positively and negatively worded statements, or repeatedly cycling through response options in a fixed sequence, such as 1–2–3–4–5.

## 3. Results

### 3.1. Sociodemographic Characteristics

A total of 654 questionnaires were returned. Following data‐quality screening, 64 questionnaires were excluded: 31 because of a completion time of less than 100 s, 14 because of logical inconsistencies between age and years of work in TCM specialty nursing, and 19 because of patterned responding. The remaining 590 questionnaires, representing 90.21% of the returned questionnaires, were included in the final analysis. The site‐specific numbers of returned and included questionnaires, together with the questionnaire‐screening process and reasons for exclusion, are presented in Figure 1. The mean age of the participants was 31.81 ± 6.73 years. The sociodemographic characteristics of the participants are shown in Table 1.

**FIGURE 1:**
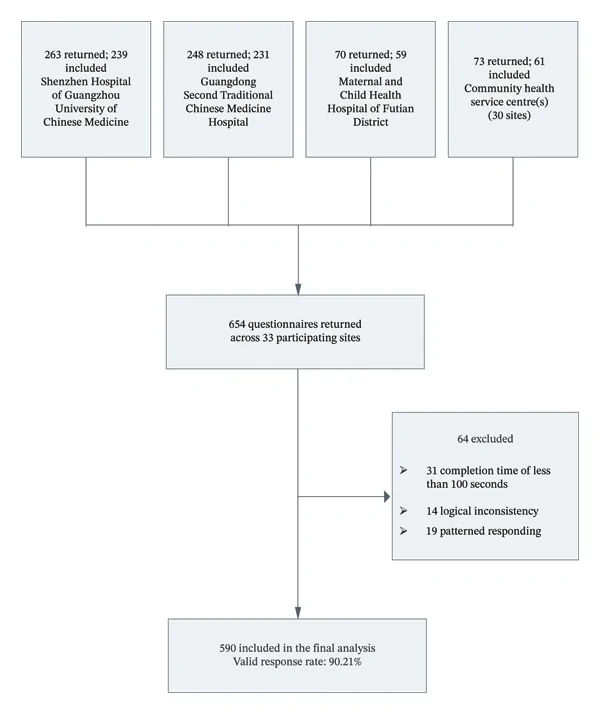
Flow diagram of participant recruitment, questionnaire screening, and inclusion in the final analysis.

**TABLE 1:** Sociodemographic characteristics (*n* = 590).

Characteristics	Classification	Number	Constituent ratio (%)
Age	29 years or younger	256	43.39
	30–39 years	241	40.85
	40 years or older	93	15.76

Gender	Female	502	85.08
	Male	88	14.92

Systematic TCM nursing training	No	53	8.98
	Yes	537	91.02

Years of TCM specialist nursing experience	1–5 years	247	41.86
	6–10 years	163	27.63
	11 years or more	180	30.51

Education	Secondary vocational education	20	3.39
	Junior college	118	20.00
	Bachelor	401	67.97
	Master’s degree and above	51	8.64

Professional title	Nurse	273	46.27
	Senior nurse	161	27.29
	Nurse in charge	127	21.53
	Associate chief nurse and above	29	4.92

TCM nursing professional level	N0‐N1 (junior)	261	44.24
	N2 (intermediate)	178	30.17
	N3‐N4 (senior)	151	25.59

Frequency of using intelligent devices in TCM nursing	1–2 times per week	191	32.37
	3–4 times per week	168	28.47
	5–6 times per week	149	25.25
	Regularly used as routine practice	82	13.90

Self‐rated familiarity with AI	Slightly familiar	146	24.75
	Moderately familiar	221	37.46
	Fairly familiar	144	24.41
	Extremely familiar	79	13.39

AI‐related training experience	No	192	32.54
	Yes	398	67.46

*Note:* TCM nursing professional level was classified according to the clinical ladder system used in the participating hospitals: N0–N1 as junior, N2 as intermediate, and N3–N4 as senior. Self‐rated familiarity with AI refers to participants’ self‐reported familiarity with AI‐related concepts and applications.

Abbreviation: TCM, Traditional Chinese Medicine.

### 3.2. Scores of Main Variables

The mean score of the Attitude Scale toward the Use of Artificial Intelligence Technologies in Nursing among TCM nurses was 48.13 ± 13.49. The mean scores for perceived organizational support, general self‐efficacy, and technology anxiety were 49.50 ± 11.26, 31.21 ± 6.59, and 23.94 ± 8.35, respectively.

Cronbach’s *α* for the attitude scale was 0.971; the corresponding coefficients for the six reverse‐scored negative‐attitude items and the nine positive‐attitude items were 0.930 and 0.951, respectively. In the derivation subset, standardized factor loadings ranged from 0.817 to 0.855; CR was 0.972, and AVE was 0.701. In the validation subset, the fixed‐parameter model yielded robust *χ* ^2^ (133) = 180.388, *p* = 0.004, CFI = 0.988, TLI = 0.991, RMSEA = 0.034 (90% CI [0.020, 0.046]), and SRMR = 0.055. For the Technology Anxiety Scale, Cronbach’s *α* was 0.936 for the total scale and 0.851, 0.869, and 0.827 for technology‐related tension, fear of technology, and privacy and security concerns, respectively. The three‐factor WLSMV model yielded scaled *χ* ^2^ (62) = 155.712, *p* < 0.001, robust CFI = 0.968, robust TLI = 0.960, robust RMSEA = 0.066 (90% CI [0.053, 0.080]), and SRMR = 0.032. Standardized factor loadings ranged from 0.704 to 0.839, and factor correlations ranged from 0.819 to 0.936.

### 3.3. Latent Profile Analysis and Profile Labeling of Attitudes Toward AI Applications

As shown in Table 2, the AIC, BIC, and aBIC values decreased progressively as the number of profiles increased from one to five. The adjusted LMR‐LRT supported the two‐profile solution over the one‐profile solution (*p* < 0.001) but did not indicate a statistically significant improvement of the three‐profile solution over the two‐profile solution (*p* = 0.113). In contrast, the BLRT supported the three‐profile solution over the two‐profile solution (*p* < 0.001). Compared with the two‐profile solution, the three‐profile solution showed substantially lower AIC, BIC, and aBIC values and identified a sizeable intermediate profile rather than only a broad low–high distinction.

**TABLE 2:** Latent profile model fit indices comparison (*n* = 590).

Model	AIC	BIC	aBIC	Entropy	Adjusted LMR *P*	BLRT *P*	Estimated profile proportions
1‐profile	26321.064	26452.468	26357.228	—	—	—	100.00%
2‐profile	20998.014	21199.500	21053.465	0.961	< 0.001	< 0.001	49.34%/50.66%
3‐profile	19359.799	19631.366	19434.537	0.939	0.113	< 0.001	25.14%/39.68%/35.18%
4‐profile	18579.337	18920.986	18673.362	0.936	0.089	< 0.001	15.83%/32.24%/29.24%/22.69%
5‐profile	18318.920	18730.652	18432.233	0.912	0.069	< 0.001	12.87%/19.91%/23.23%/25.49%/18.50%

The three‐profile solution demonstrated high classification quality, with an entropy value of 0.939 and diagonal average posterior probabilities of 0.967, 0.972, and 0.977 for Profiles 1, 2, and 3, respectively. The complete average posterior probability matrix is presented in Supporting File 8. The estimated profile proportions were 25.14%, 39.68%, and 35.18%, with no profile representing a small proportion of the sample. Although the AIC, BIC, and aBIC values continued to decrease for the four‐ and five‐profile solutions, their adjusted LMR‐LRT results remained nonsignificant (*p* = 0.089 and *p* = 0.069, respectively), and entropy decreased to 0.936 and 0.912. Inspection of the item‐level profile plots showed that the additional profiles in the four‐ and five‐profile solutions mainly subdivided the same ordered low‐to‐high attitude gradient and displayed largely parallel item‐response patterns rather than substantively distinct configurations. Considering the statistical fit, classification quality, profile size, parsimony, and substantive interpretability, the three‐profile solution was retained as the most parsimonious and interpretable representation of the data. The observed means showed a consistent ordered gradient across all 15 attitude items (Figure 2). The low AI acceptance profile had the lowest observed item means (1.78–2.23), the moderate AI acceptance profile had intermediate means (2.91–3.37), and the high AI acceptance profile had the highest means (3.91–4.42). The broadly similar response shapes indicate that the profiles primarily differed in overall AI acceptance level rather than in qualitatively distinct item‐specific patterns (Table 3).

**FIGURE 2:**
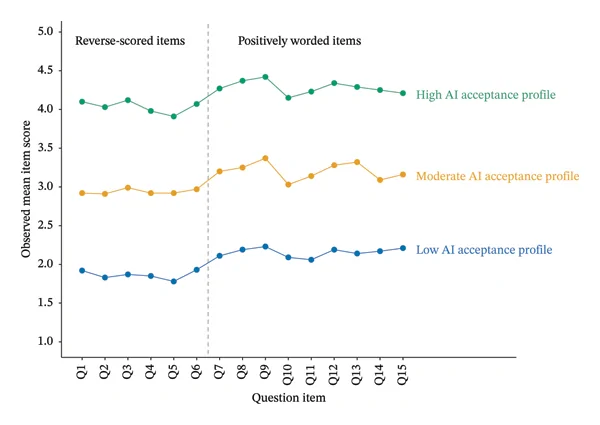
Item score characteristics across latent profiles of attitudes toward AI applications among TCM nurses.

**TABLE 3:** Item‐level mean scores of attitudes toward AI applications among TCM nurses.

	Profile 1: Low AI acceptance profile	Profile 2: Moderate AI acceptance profile	Profile 3: High AI acceptance profile
Q1	1.92 ± 0.72	2.92 ± 0.70	4.10 ± 0.65
Q2	1.83 ± 0.71	2.91 ± 0.68	4.03 ± 0.74
Q3	1.87 ± 0.61	2.99 ± 0.64	4.12 ± 0.71
Q4	1.85 ± 0.65	2.92 ± 0.66	3.98 ± 0.63
Q5	1.78 ± 0.68	2.92 ± 0.67	3.91 ± 0.71
Q6	1.93 ± 0.71	2.97 ± 0.62	4.07 ± 0.65
Q7	2.11 ± 0.67	3.20 ± 0.64	4.27 ± 0.64
Q8	2.19 ± 0.69	3.25 ± 0.69	4.37 ± 0.61
Q9	2.23 ± 0.71	3.37 ± 0.72	4.42 ± 0.59
Q10	2.09 ± 0.72	3.03 ± 0.69	4.15 ± 0.66
Q11	2.06 ± 0.70	3.14 ± 0.61	4.23 ± 0.74
Q12	2.19 ± 0.75	3.28 ± 0.69	4.34 ± 0.65
Q13	2.14 ± 0.60	3.32 ± 0.65	4.29 ± 0.63
Q14	2.17 ± 0.71	3.09 ± 0.65	4.25 ± 0.67
Q15	2.21 ± 0.70	3.16 ± 0.69	4.21 ± 0.70

*Note:* Values are presented as observed means ± standard deviations calculated according to participants’ most‐likely profile assignment. These values are descriptive, whereas Figure 2 presents the observed profile means.

### 3.4. Sensitivity Analysis of Local Independence

In the targeted sensitivity analysis, the residual covariances between Q1 and Q4, Q2 and Q6, and Q7 and Q15 were freely estimated and constrained to be equal across profiles. The unstandardized residual covariance estimates were 0.141, 0.131, and 0.124, respectively (all *p* < 0.001). The estimated profile proportions were 24.26%, 39.33%, and 36.41%, compared with 25.14%, 39.68%, and 35.18% in the primary model. Entropy remained high (0.934 vs. 0.939), and the diagonal average posterior probabilities were 0.970, 0.964, and 0.972 for Profiles 1, 2, and 3, respectively. The sensitivity model retained the same ordered low‐to‐high item‐response pattern. Comparison of modal classifications showed 97.46% agreement (575/590) with the primary model, with an adjusted Rand index of 0.923. These findings support the robustness of the retained three‐profile solution to the local‐dependence specification examined.

### 3.5. Univariate Analysis of Latent Profiles of Attitudes Toward AI Applications Among TCM Nurses

The univariate analysis showed statistically significant differences across latent profiles in years of TCM specialist nursing experience, professional title, TCM nursing professional level, frequency of using intelligent devices in TCM nursing, self‐rated familiarity with AI, and AI‐related training experience (all *p* < 0.001). Technology anxiety also differed significantly across profiles (*p* = 0.016). Details are shown in Table 4.

**TABLE 4:** Univariate comparisons of participant characteristics across latent profiles of attitudes toward artificial intelligence applications among traditional Chinese medicine nurses (*N* = 590).

Variable	Category	Profile 1 low AI acceptance (*n* = 151)	Profile 2 moderate AI acceptance (*n* = 231)	Profile 3 high AI acceptance (*n* = 208)	Test statistic (df)	*p* value
Age		31.00 (27.00, 35.50)	31.00 (26.00, 37.00)	31.00 (26.00, 36.00)	*H* (2) = 0.12	0.944

Age group	29 years or younger	64 (42.4)	101 (43.7)	91 (43.8)	*χ* ^2^ (4) = 0.96	0.916
	30–39 years	62 (41.1)	91 (39.4)	88 (42.3)		
	40 years or older	25 (16.6)	39 (16.9)	29 (13.9)		

Gender	Female	128 (84.8)	197 (85.3)	177 (85.1)	*χ* ^2^ (2) = 0.02	0.991
	Male	23 (15.2)	34 (14.7)	31 (14.9)		

Systematic TCM nursing training	No	10 (6.6)	22 (9.5)	21 (10.1)	*χ* ^2^ (2) = 1.43	0.490
	Yes	141 (93.4)	209 (90.5)	187 (89.9)		

Years of TCM specialist nursing experience	1–5 years	109 (72.2)	105 (45.5)	33 (15.9)	*χ* ^2^ (4) = 143.61	< 0.001
	6–10 years	28 (18.5)	75 (32.5)	60 (28.8)		
	11 years or more	14 (9.3)	51 (22.1)	115 (55.3)		

Education	Secondary vocational education	4 (2.6)	6 (2.6)	10 (4.8)	*χ* ^2^ (6) = 5.50	0.481
	Junior college	29 (19.2)	42 (18.2)	47 (22.6)		
	Bachelor	101 (66.9)	163 (70.6)	137 (65.9)		
	Master’s degree and above	17 (11.3)	20 (8.7)	14 (6.7)		

Professional title	Nurse	97 (64.2)	109 (47.2)	67 (32.2)	*χ* ^2^ (6) = 57.79	< 0.001
	Senior nurse	41 (27.2)	65 (28.1)	55 (26.4)		
	Nurse in charge	9 (6.0)	50 (21.6)	68 (32.7)		
	Associate chief nurse and above	4 (2.6)	7 (3.0)	18 (8.7)		

TCM nursing professional level	N0‐N1 (junior)	101 (66.9)	112 (48.5)	48 (23.1)	*χ* ^2^ (4) = 85.66	< 0.001
	N2 (Intermediate)	38 (25.2)	69 (29.9)	71 (34.1)		
	N3‐N4 (Senior)	12 (7.9)	50 (21.6)	89 (42.8)		

Frequency of using intelligent devices in TCM nursing	1–2 times per week	80 (53.0)	71 (30.7)	40 (19.2)	*χ* ^2^ (6) = 68.37	< 0.001
	3–4 times per week	36 (23.8)	81 (35.1)	51 (24.5)		
	5–6 times per week	25 (16.6)	56 (24.2)	68 (32.7)		
	Regularly used as routine practice	10 (6.6)	23 (10.0)	49 (23.6)		

Self‐rated familiarity with AI	Slightly familiar	63 (41.7)	65 (28.1)	18 (8.7)	*χ* ^2^ (6) = 109.08	< 0.001
	Moderately familiar	61 (40.4)	101 (43.7)	59 (28.4)		
	Fairly familiar	23 (15.2)	43 (18.6)	78 (37.5)		
	Extremely familiar	4 (2.6)	22 (9.5)	53 (25.5)		

AI‐related training experience	No	84 (55.6)	78 (33.8)	30 (14.4)	*χ* ^2^ (2) = 67.93	< 0.001
	Yes	67 (44.4)	153 (66.2)	178 (85.6)		

Technology anxiety		21.00 (19.00, 23.00)	22.00 (20.00, 26.00)	21.00 (20.00, 24.00)	*H* (2) = 8.24	0.016

Perceived organizational support		52.00 (49.50, 56.00)	52.00 (48.00, 56.00)	52.00 (48.00, 55.00)	*H* (2) = 0.57	0.751

General self‐efficacy		34.00 (30.00, 35.00)	34.00 (30.00, 35.00)	33.00 (29.75, 35.00)	*H* (2) = 0.19	0.908

*Note:* Values are presented as *n* (%) or median (first quartile, third quartile). Profile membership was based on most‐likely‐class assignment; therefore, profile sizes may differ slightly from the observed proportions. *χ* ^2^ denotes the Pearson chi‐square statistic, and *H* denotes the Kruskal–Wallis statistic. These comparisons were descriptive only and were not used for R3STEP covariate selection.

### 3.6. Factors Associated With Latent Profile Membership

Factors associated with latent profile membership were examined using the bias‐adjusted R3STEP procedure. All 13 covariates were entered simultaneously, with the low AI acceptance profile, Profile 1, as the reference category. VIF‐equivalent values ranged from 1.027 to 2.527, indicating no evidence of problematic multicollinearity among the covariates, as shown in Supporting File 7. The results indicated that years of TCM specialist nursing experience, education, TCM nursing professional level, professional title, self‐rated familiarity with AI, AI‐related training experience, intelligent‐device‐use frequency, and technology anxiety were associated with profile membership. Longer TCM specialist nursing experience, AI‐related training experience, and higher self‐rated familiarity with AI were associated with higher odds of membership in the high AI acceptance profile. Technology anxiety was associated with higher odds of Profile 2 versus Profile 1 membership (aOR = 1.044, *p* = 0.023); the Profile 3 versus Profile 1 comparison was not statistically significant (*p* = 0.434). Detailed results are presented in Table 5. The key associations remained consistent in direction and statistical significance in the theory‐informed core‐covariate R3STEP sensitivity analysis reported in Supporting File 8.

**TABLE 5:** Associations between covariates and latent profile membership using bias‐adjusted three‐step multinomial logistic regression (R3STEP; *n* = 590).

Variable/comparison	Profile 2 vs profile 1, aOR (95% CI)	*p* value	Profile 3 vs profile 1, aOR (95% CI)	*p* value
Age, per year	1.005 (0.966–1.045)	0.818	1.015 (0.969–1.064)	0.528
Sex: male vs female	0.985 (0.488–1.989)	0.967	1.019 (0.439–2.364)	0.965
Systematic TCM nursing training: yes vs no	0.713 (0.285–1.785)	0.470	1.386 (0.467–4.112)	0.556

*TCM specialist nursing experience (ref: 1–5 years)*				
6–10 years	2.461 (1.324–4.577)	0.004	5.331 (2.418–11.755)	< 0.001
≥ 11 years	2.889 (1.276–6.540)	0.011	15.394 (5.969–39.702)	< 0.001
Education: bachelor’s degree or above vs junior college or below	0.921 (0.527–1.609)	0.771	0.433 (0.210–0.892)	0.023

*Professional title (ref: nurse)*				
Senior nurse	1.653 (0.941–2.903)	0.080	1.541 (0.749–3.169)	0.240
Nurse in charge or above	3.706 (1.509–9.101)	0.004	7.692 (2.847–20.784)	< 0.001

*TCM nursing professional level (ref: N0–N1)*				
N2	1.616 (0.909–2.874)	0.102	3.483 (1.655–7.327)	0.001
N3–N4	2.786 (1.221–6.357)	0.015	9.241 (3.623–23.568)	< 0.001

*Intelligent-device use (ref: 1–2 times/week)*				
3–4 times/week	2.692 (1.469–4.934)	0.001	2.516 (1.129–5.607)	0.024
5–6 times/week	1.980 (1.005–3.901)	0.048	2.526 (1.087–5.870)	0.031
Routine use	1.783 (0.674–4.720)	0.244	5.017 (1.657–15.187)	0.004

*Self-rated familiarity with AI (ref: slightly familiar)*				
Moderately familiar	1.632 (0.932–2.859)	0.087	3.596 (1.501–8.612)	0.004
High familiarity (fairly or extremely familiar)	2.013 (1.010–4.011)	0.047	13.988 (5.643–34.675)	< 0.001
AI training: yes vs no	2.106 (1.278–3.469)	0.003	4.937 (2.445–9.968)	< 0.001
Technology anxiety, per point	1.044 (1.006–1.083)	0.023	1.019 (0.972–1.068)	0.434
Perceived organizational support, per point	1.001 (0.968–1.036)	0.951	1.019 (0.981–1.058)	0.339
General self‐efficacy, per point	1.008 (0.955–1.064)	0.780	0.948 (0.888–1.011)	0.103

*Note:* Profile 1 (low AI acceptance profile) was the reference profile. All covariates were entered simultaneously. High AI familiarity combined the original fairly familiar and extremely familiar categories. *p* values are from the R3STEP logit coefficients.

Abbreviations: aOR, adjusted odds ratio; CI, confidence interval.

## 4. Discussion

### 4.1. Summary of Main Findings

This study used LPA to examine latent differences in attitudes toward AI applications among TCM nurses and to identify factors associated with different attitude profiles. The results showed that the mean total score for attitudes toward AI applications among TCM nurses was 48.13 ± 13.49, suggesting a moderate level overall. This score was lower than that reported by Hu et al. [43] among general clinical nurses, suggesting that TCM nurses may be more cautious in their acceptance of AI technologies. LPA identified three profiles: the low AI acceptance profile (25.14%), the moderate AI acceptance profile (39.68%), and the high AI acceptance profile (35.18%). The profiles showed similar patterns across the 15 items and differed primarily in overall AI acceptance level. Years of TCM specialist nursing experience, education, professional title, TCM nursing professional level, frequency of using intelligent devices in TCM nursing, self‐rated familiarity with AI, and AI‐related training experience were associated with profile membership. Technology anxiety was also associated with higher odds of membership in the moderate rather than the low AI acceptance profile. Age, sex, TCM nursing training, perceived organizational support, and general self‐efficacy were not significantly associated with profile membership in either comparison using Profile 1 as the reference.

### 4.2. Interpretation of Latent Profile Patterns

The three latent profiles identified in this study indicate clear differences in attitudes toward AI applications among TCM nurses. The low AI acceptance profile had relatively low scores across all 15 items, which may reflect a more conservative attitude toward AI and limited recognition of its value, usability, and clinical compatibility with TCM nursing. The moderate AI acceptance profile had intermediate scores, suggesting that these nurses may recognize some potential value of AI while still considering whether it can meet the needs of TCM nursing, such as holistic assessment, syndrome‐based nursing, and individualized intervention. The high AI acceptance profile had higher scores, indicating a greater tendency to accept AI from the perspectives of improving efficiency, standardizing workflows, supporting syndrome assessment, and enhancing nursing quality.

### 4.3. Educational Attainment in Relation to AI Acceptance

The results indicated that educational attainment was associated with latent profile membership. Compared with nurses with a junior college education or below, those with a bachelor’s degree or above had lower relative odds of belonging to the high rather than the low AI acceptance profile. This finding contrasts with that of Wei et al. [44], who reported that nurses with a master’s degree or above were more likely to belong to the positive acceptance profile than to the cautious skepticism profile. In a sample of general clinical nurses, Li et al. found that the high AI acceptance–utility‐oriented profile was characterized by higher perceived AI quality and personal utility and was interpreted as greater recognition of AI’s potential to improve work efficiency and optimize nursing workflows [45]. The discrepant finding in the present study may reflect the professional context of TCM nursing. TCM nursing is grounded in a holistic approach and syndrome differentiation–based nursing [46], while TCM syndrome differentiation draws on information obtained through inspection, auscultation‐olfaction, inquiry, and palpation [47]. Accordingly, when appraising AI, TCM nurses may consider not only its capacity to support work efficiency and nursing workflows but also whether its recommendations can be integrated with individualized syndrome differentiation–based nursing while preserving professional judgment and humanistic care. A Chinese survey that included nurses found that respondents were generally positive toward AI in nursing but also identified concerns regarding system quality, attribution of medical responsibility, patient privacy, and potential alienation of the nurse–patient relationship [6]. In this context, higher educational attainment may not necessarily correspond to greater AI acceptance among TCM nurses; nurses with higher educational attainment may place a greater emphasis on whether AI can reliably support TCM nursing judgment and align with syndrome differentiation–based nursing.

### 4.4. Professional Characteristics and AI Attitude Profiles

The R3STEP results indicated that years of TCM specialist nursing experience, education, professional title, and TCM nursing professional level were associated with latent profile membership of attitudes toward AI applications among TCM nurses. Compared with nurses with shorter TCM specialist nursing experience, those with longer experience had higher odds of membership in the moderate and high AI acceptance profiles. This finding is not entirely consistent with the study by Nirgiz et al. [48], which reported that nurses with 0–3 years of work experience had relatively higher positive‐attitude scores toward AI. This difference may be related to differences in study populations, professional contexts, and technology exposure. General clinical nurses with shorter work experience may be more accustomed to digital environments and more receptive to emerging technologies [49]. In contrast, attitudes toward AI applications among TCM nurses may be more closely related to TCM nursing experience, specialist technical competence, and understanding of clinical contexts. TCM nursing is centered on holism and syndrome‐based nursing. It requires nurses to make comprehensive judgments by integrating patients’ constitution, symptoms, emotional status, and treatment responses, which makes TCM nursing highly experiential, comprehensive, and context‐dependent [50, 51]. Within the present sample, longer working experience, higher professional titles, and higher professional levels characterized profiles that placed greater value on the potential role of AI in integrating four‐diagnostic information, supporting dynamic syndrome assessment, informing individualized nursing plans, and standardizing appropriate TCM nursing techniques.

### 4.5. Self‐Rated Familiarity With AI, Training, and Device Exposure

This study found that self‐rated familiarity with AI, AI‐related training experience, and frequency of intelligent‐device use were associated with AI attitude‐profile membership among TCM nurses. Higher self‐rated familiarity with AI was associated with higher odds of membership in profiles characterized by more positive attitudes toward AI applications, which is consistent with the findings of Ramadan et al. [52]. This finding suggests that greater familiarity with AI may coincide with less uncertainty and a more balanced appraisal of its potential value and risks, rather than simple acceptance or rejection [53]. The results also showed that nurses who had received AI‐related training were more likely to belong to the moderate AI acceptance profile and the high AI acceptance profile. This pattern may reflect a clearer understanding of the technical principles, application value, and practical boundaries of AI among nurses with AI‐related training experience. Such training may also provide opportunities for nurses to develop more informed perceptions of the advantages, clinical compatibility, and potential risks of AI through practical operation, case discussion, and scenario‐based demonstration, which may be accompanied by fewer concerns and less resistance arising from unfamiliarity [54, 55]. The studies by Karaarslan et al. [56] and Shokr [57] provide support for this interpretation. The frequency of using intelligent devices in TCM nursing was also associated with profile membership, especially in the high AI acceptance profile. Compared with nurses who used such devices 1–2 times per week, those who used them 3–4 times per week, 5–6 times per week, or as part of routine practice had higher odds of membership in the high AI acceptance profile. This finding suggests that more frequent hands‐on experience with intelligent devices may be closely related to greater AI acceptance among TCM nurses. Oweidat et al. [58] also emphasized the importance of practical experience with AI among general clinical nurses. Overall, greater self‐rated familiarity with AI, AI‐related training experience, and experience with intelligent devices may be associated with greater familiarity with AI technologies, lower uncertainty in application, and a stronger sense of control over technological systems, which may correspond to more positive attitudes toward AI applications [59].

### 4.6. Technology Anxiety and Practice Implications

It is worth noting that the relationship between technology anxiety and latent profiles of attitudes toward AI applications was not simply linear in this study. The univariate analysis showed that the moderate AI acceptance profile had the highest technology anxiety score [22.00 (20.00, 26.00)], whereas the low AI acceptance profile [21.00 (19.00, 23.00)] and the high AI acceptance profile [21.00 (20.00, 24.00)] had relatively lower scores. Higher technology anxiety was associated with greater odds of membership in Profile 2 than in Profile 1, whereas the association was not significant for Profile 3 versus Profile 1. This pattern suggests that technology anxiety does not simply reflect rejection of AI; among nurses in the moderate AI acceptance profile, the recognition of AI’s potential value appeared to coexist with unresolved concerns [60, 61]. Nurses in the moderate AI acceptance profile may have recognized the potential value of AI in improving nursing efficiency, integrating clinical information, and optimizing workflows. However, they may still have uncertainty regarding technological reliability, compatibility with TCM nursing, responsibility boundaries, and ethical risks. Therefore, these nurses may not simply reject or resist AI. Rather, while recognizing its application value, they may continue to carefully evaluate its clinical applicability, implementation conditions, and potential risks [62]. The coexistence of perceived value and unresolved concerns may be closely related to the higher level of technology anxiety observed in this profile. In contrast, nurses in the low AI acceptance profile may have had limited cognitive engagement with AI technologies and limited participation in AI‐related applications. Lower attitudes toward AI applications may not necessarily be accompanied by higher technology anxiety [63]. The high AI acceptance profile was also characterized by higher self‐rated familiarity with AI, more AI‐related training experience, and more frequent use of intelligent devices. This profile may also reflect a clearer understanding of the boundaries and clinical value of AI applications, which is consistent with the nonsignificant association observed between technology anxiety and membership in this profile. In this study, neither perceived organizational support nor general self‐efficacy was significantly associated with profile membership in either comparison using Profile 1 as the reference. From the perspective of the TAM, general confidence and workplace support may not fully capture nurses’ specific judgments about the usefulness and ease of use of AI in TCM nursing. Future studies could therefore include AI‐specific organizational support and AI self‐efficacy to better understand how these factors relate to attitudes toward AI applications.

### 4.7. Nursing Implications for AI Implementation in TCM Nursing

The findings of this study have practical implications for TCM nursing management. First, AI implementation in TCM nursing may benefit from moving beyond uniform training or one‐way technical education. Instead, stratified, scenario‐based, and practice‐oriented management strategies could be considered according to different attitude profiles among nurses [21, 56]. For nurses in the low AI acceptance profile, training could focus on basic knowledge, safety boundaries, and trust building. Clinical examples can be used to illustrate the specific roles of AI in four‐diagnostic information collection, constitution identification, humanistic care, and health education. Training could also clarify that AI is intended to support rather than replace nurses’ syndrome‐based judgment, emotional care, and humanistic communication. Such clarification may help position AI as a supportive tool for nursing decision‐making and workflow optimization, with its application viewed from a more open and inclusive perspective that recognizes the professional value, clinical judgment, and humanistic role of TCM nurses. For nurses in the moderate AI acceptance profile, scenario‐based training can be designed around typical TCM nursing tasks. Examples include standardized collection of tongue appearance, complexion, pulse, and symptom information; nursing application of constitution identification results; recommendation of syndrome‐based nursing plans for patients with different syndrome patterns; quality control of appropriate TCM nursing techniques such as moxibustion, acupoint application, and auricular acupoint therapy; and generation of TCM health education content. Scenario‐based training may be aligned with familiar TCM nursing tasks, with particular attention to nurses’ concerns regarding the clinical value, reliability, compatibility, and potential risks of AI. For nurses in the high AI acceptance profile, nursing managers may further support their exemplary and leading roles. These nurses can be encouraged to participate in the development of AI application standards, operating procedures, and quality evaluation criteria for TCM nursing. They may also help teams assess whether AI‐generated outputs are consistent with the principles of syndrome‐based nursing and the practical needs of TCM nursing. This may support the transition of AI applications from individual attempts to standardized team‐level practice.

### 4.8. Limitations and Future Research

This study has several limitations. First, the cross‐sectional design limits interpretation of the observed relationships to associations. Because self‐rated familiarity with AI, AI‐related training experience, frequency of intelligent‐device use, technology anxiety, and AI attitudes were assessed concurrently, their temporal ordering remains unclear, and some of these relationships may be reciprocal. Second, convenience sampling was used, and participants were mainly recruited from multiple TCM hospitals and affiliated community health service institutions in South China. Therefore, the generalizability of the findings to other regions or hospitals of different levels requires further validation. Third, this study mainly included general perceived organizational support and general self‐efficacy. Fourth, the device‐use and AI familiarity items had measurement limitations. The device‐use options combined weekly frequency categories with a description of routine use and did not include options for no use or less‐than‐weekly use. Self‐rated familiarity with AI was assessed using a single item without explicit descriptive anchors or a “Not at all familiar” option. These response options may have led to misclassification, particularly among respondents with infrequent device use or no familiarity with AI. Additionally, self‐rated familiarity may be affected by self‐assessment bias and may not fully correspond to objectively assessed knowledge. These limitations should be considered when interpreting associations involving these variables.

Future studies may further examine AI‐specific support, AI self‐efficacy, and AI literacy to provide a more comprehensive explanation of differences in attitudes toward AI applications among TCM nurses. Future research may conduct longitudinal studies across different regions and different levels of medical institutions. Data on nurses’ actual frequency of AI system use, training effectiveness, and frequency of using intelligent devices in TCM nursing could also be incorporated to further examine differences in TCM nurses’ cognition, adaptation, and use of AI technologies. In addition, qualitative interview data may be used to better understand the practical needs and related factors of AI applications in TCM nursing contexts, providing evidence for more targeted AI training and nursing management strategies.

## 5. Conclusion

This multicenter cross‐sectional study identified three latent profiles of attitudes toward AI applications among TCM nurses: the low AI acceptance profile, the moderate AI acceptance profile, and the high AI acceptance profile. These findings indicate that TCM nurses’ attitudes toward AI applications are not homogeneous and that a person‐centered approach may provide a more nuanced understanding than total scale scores alone. Profile membership was associated with several occupational and technology‐related characteristics, including TCM specialist nursing experience, education, professional title, TCM nursing professional level, intelligent‐device use, self‐rated familiarity with AI, AI‐related training, and technology anxiety; these associations varied across profile comparisons. These findings suggest that attitudes toward AI applications in TCM nursing are closely related to both professional experience and technology‐related exposure. In practice, these findings may inform consideration of differences in nurses’ attitude profiles and the development of stratified, scenario‐based, and practice‐oriented AI implementation strategies in TCM nursing. Future longitudinal and mixed‐methods studies are needed to examine the stability and changes of these profiles over time and to further explore how AI‐related training, clinical experience, and organizational support may facilitate the integration of AI into TCM nursing practice.

**Nomenclature**
AI: Artificial intelligence
TCM: Traditional Chinese medicine
TAM: Technology acceptance model
LPA: Latent profile analysis
STROBE: Strengthening the Reporting of Observational Studies in Epidemiology
AIC: Akaike information criterion
BIC: Bayesian information criterion
aBIC: sample‐size–adjusted Bayesian information criterion
LMR: Lo–Mendell–Rubin likelihood ratio test
BLRT: bootstrap likelihood ratio test
OR: odds ratio
CI: confidence interval
VIF: variance inflation factor

## Author Contributions

Shuang Lu and Xian Liu contributed equally to this work. Shuang Lu designed the study, collected and analyzed the data, and drafted the manuscript. Xian Liu contributed to study design, data analysis, interpretation of the results, and manuscript revision. Longxian Liu assisted with data collection and data management. Xinrong Tan, Guo Yu, Wenxia Hu, Meigui Peng, Ziyi Yuan, and Shaoyang Cui assisted with questionnaire distribution and data collection. Zining Guo and Meng Wu supervised the study, contributed to the interpretation of the results, and critically revised the manuscript.

## Funding

This research was funded by the 2025 Special Project for Basic Research of Shenzhen City under the Natural Science Fund Scheme (Grant No. JCYJ20250604190224031).

## Disclosure

The funder had no role in the study design, data collection, data analysis, manuscript preparation, or decision to submit the manuscript for publication. All authors read and approved the final manuscript.

## Ethics Statement

Ethical approval was obtained from the Ethics Committee of Shenzhen Hospital of Guangzhou University of Chinese Medicine (Futian) on 9 January 2026 (Approval No. GZYLL(KY)‐2026‐003), before participant recruitment and data collection commenced. All participants provided electronic informed consent before completing the anonymous questionnaire.

## Conflicts of Interest

The authors declare no conflicts of interest.

## Supporting Information

Additional supporting information can be found online in the Supporting Information section.

## Supporting information


**Supporting Information 1** Supporting File 1. General information questionnaire.

**Supporting Information 2** Supporting File 2. Variable coding scheme.

**Supporting Information 3** Supporting File 3. Ethics approval document.

**Supporting Information 4** Supporting File 4. Participating community health service centers and stations.

**Supporting Information 5** Supporting File 5. Measurement instruments and psychometric properties.

**Supporting Information 6** Supporting File 6. Categorical covariate recoding for R3STEP analysis.

**Supporting Information 7** Supporting File 7. Collinearity diagnostics.

**Supporting Information 8** Supporting File 8. Classification quality and sensitivity analyses.

**Supporting Information 9** Supporting File 9. STROBE checklist.

## Data Availability

The datasets used and/or analyzed during the current study are available from the corresponding authors upon reasonable request.
