# Supplementary material for: Latent Profiles of Attitudes Toward Artificial Intelligence Applications Among Traditional Chinese Medicine Nurses and Associated Factors: A Multicenter Cross‐Sectional Study
Source: J Nurs Manag. 2026 Nov 4;2026:6814652. doi: 10.1155/jonm/6814652 (PMC13735534; doi:10.1155/jonm/6814652)
Supplement: Supplementary file 1 [file JONM-2026-6814652-s002.pdf]

文件编码：F-GZSY-LL-GZCX-14-2-1

广州中医药大学深圳医院（福田）医学伦理委员会

伦理审查批件

批件号：GZYLL(KY)-2026-003

项目名称	基于知识图谱的颈椎病数智化防控管理系统的构建与应用		
项目来源	2025 年度深圳市自然科学基金项目		
项目负责人	吴蒙	所在科室	康复科
申办单位/组长单位	无	CRO	无
审查文件	1.初始审查申请表；<br>2.研究方案（版本号 1.0，版本日期：2025 年 12 月 3 日）；<br>3.知情同意书（版本号 1.0，版本日期：2025 年 12 月 3 日）；<br>4 主要研究者简历，研究人员职责表，研究者经济利益声明；<br>5.科研立项通知。		
审查类别	初始审查		
审查方式	会议审查		
审查日期	2026 年 1 月 8 日		
审查地点	广州中医药大学深圳医院		
审查意见	根据国家卫生健康委《涉及人的生命科学和医学研究伦理审查办法》（2023 年），国家药监局、国家卫生健康委《药物临床试验质量管理规范》（2020 年）、《医疗器械临床试验质量管理规范》（2022 年），《科技伦理审查办法（试行）》（2023 年），WMA《赫尔辛基宣言》和 CIOMS《人体生物医学研究国际道德指南》的伦理原则。经本伦理委员会审查，同意按所批准的临床研究方案、知情同意书开展本项研究。<br>注：<br>1、请遵循 GCP 原则、遵循伦理委员会批准的方案开展临床研究，保护受试者的健康和权利。<br>2、对研究方案、知情同意书、招募材料等的任何修改，请提交修正案审查申请。<br>3、发生 SAE，请及时提交严重不良事件报告。<br>4、如有不依从/违背方案的情况，请及时提交违背方案报告。<br>5、请根据年度/定期跟踪审查频率，及时提交研究进展报告。<br>6、暂停或提前终止临床研究，请及时提交暂停/终止研究报告。<br>7、完成临床研究，请提交结题报告。		

文件编码：F-GZSY-LL-GZCX-14-2-1

广州中医药大学深圳医院（福田）医学伦理委员会

伦理审查批件

年度定期/跟踪审查频率	12个月<br>预计审查日期：2027年1月9日	批件有效期	自 2026年1月9日起<br>至 2027年1月9日止
联系人（委员会秘书）	卢琳	联系电话	0755-83548506
主任委员（被授权者）签名	广州中医药大学深圳医院（福田）医学伦理委员会（盖章）<br>2026年1月9日		
