# Supplementary material for: Latent Profiles of Attitudes Toward Artificial Intelligence Applications Among Traditional Chinese Medicine Nurses and Associated Factors: A Multicenter Cross‐Sectional Study
Source: J Nurs Manag. 2026 Nov 4;2026:6814652. doi: 10.1155/jonm/6814652 (PMC13735534; doi:10.1155/jonm/6814652)
Supplement: Supplementary file 2 [file JONM-2026-6814652-s001.docx]

Supplementary File 2. Variable Coding Scheme

The table below presents the coding scheme and reference categories for the listed variables.

| Variable | Assignment method |
| --- | --- |
| Dependent variable: latent profile class | Class 1: Low AI acceptance profile = reference; Class 2: Moderate AI acceptance profile = 1; Class 3: High AI acceptance profile = 2 |
| Age | Original value (years) |
| Sex | Female = reference；Male = 1 |
| Education | Junior college or below = reference; Bachelor’s degree or above = 1 |
| Systematic TCM nursing training | No = reference; Yes = 1 |
| Working years in TCM specialized nursing | 1–5 years = reference; 6–10 years = 1; 11 years or more = 2 |
| Professional title | Nurse = reference; Senior nurse = 1; Nurse in charge or above = 2 |
| TCM nursing professional level | N0–N1 (Junior) = reference; N2 (Intermediate) = 1; N3–N4 (Senior) = 2 |
| Frequency of using intelligent devices in TCM nursing | 1–2 times per week = reference; 3–4 times per week = 1; 5–6 times per week = 2; Regularly used as routine practice = 3 |
| Self-rated familiarity with AI | Slightly familiar = reference; Moderately familiar = 1; High familiarity (fairly familiar or extremely familiar) = 2. |
| AI-related training experience | No = reference; Yes = 1 |
| Technology anxiety | Original total score |
| Perceived organizational support | Original total score |
| General self-efficacy | Original total score |

Note: TCM = traditional Chinese medicine. All 13 candidate covariates listed above were entered simultaneously in the bias-adjusted R3STEP model. The TCM nursing professional-level categories correspond to item 7 of the questionnaire: N0–N1 (Junior), N2 (Intermediate), and N3–N4 (Senior).
