# Supplementary material for: Latent Profiles of Attitudes Toward Artificial Intelligence Applications Among Traditional Chinese Medicine Nurses and Associated Factors: A Multicenter Cross‐Sectional Study
Source: J Nurs Manag. 2026 Nov 4;2026:6814652. doi: 10.1155/jonm/6814652 (PMC13735534; doi:10.1155/jonm/6814652)
Supplement: Supplementary file 3 [file JONM-2026-6814652-s003.docx]

**Supplementary File 4. Participating Community Health Service Centers and Stations**

Note: English names are transliterations of the official Chinese site names. The 30 sites were included as a community health service network; site-specific participant numbers were not retained

| **No.** | **Participating community health service center/station** |
| --- | --- |
| 1 | Yutian Community Health Service Center |
| 2 | Fuzhong Community Health Service Center |
| 3 | Shenkang Village Community Health Service Center |
| 4 | Jinghua Community Health Service Center |
| 5 | Lianhua North Community Health Service Center (Outpatient Department) |
| 6 | Jingxin Community Health Service Center/Station |
| 7 | Tefa Community Health Service Center/Station |
| 8 | Cuihai Community Health Service Station/Center |
| 9 | Zhulin Community Health Service Station/Center |
| 10 | Lianhua Second Village Community Health Service Center |
| 11 | Jingmi Community Health Service Center |
| 12 | Jinbi Community Health Service Station |
| 13 | Xiyuan Community Health Service Station/Center |
| 14 | Shazui Community Health Service Center |
| 15 | Tian'an Community Health Service Center |
| 16 | Cuiwan Community Health Service Center |
| 17 | Nongyuan Community Health Service Station/Center |
| 18 | Xinsha Community Health Service Center |
| 19 | Xiangmi Second Village Community Health Service Center |
| 20 | Caitian Village Community Health Service Station |
| 21 | Civic Center Community Health Service Station |
| 22 | Xiang'an Community Health Service Station |
| 23 | Taiyuan Community Health Service Center |
| 24 | State Tax Bureau Community Health Service Station |
| 25 | New Generation Industrial Park Community Health Service Station |
| 26 | Hailian Community Health Service Station |
| 27 | Xinzhou Community Health Service Center |
| 28 | Shawei Community Health Service Center |
| 29 | Bihai Yuntian Community Health Service Center |
| 30 | Xiasha Community Health Service Center |
