# Supplementary material for: Latent Profiles of Attitudes Toward Artificial Intelligence Applications Among Traditional Chinese Medicine Nurses and Associated Factors: A Multicenter Cross‐Sectional Study
Source: J Nurs Manag. 2026 Nov 4;2026:6814652. doi: 10.1155/jonm/6814652 (PMC13735534; doi:10.1155/jonm/6814652)
Supplement: Supplementary file 4 [file JONM-2026-6814652-s004.docx]

**Supplementary File 6. Original response categories and analytic coding of categorical covariates used in the R3STEP model**

| **Covariate** | **Original response categories** | **Analytic categories used in R3STEP, n (%)** | **Reference category** |
| --- | --- | --- | --- |
| Sex | Female; male | Female: 502 (85.08%); male: 88 (14.92%) | Female |
| Systematic TCM nursing training | No; yes | No: 53 (8.98%); yes: 537 (91.02%) | No |
| TCM specialist nursing experience | 1–5 years; 6–10 years; ≥11 years | 1–5 years: 247 (41.86%); 6–10 years: 163 (27.63%); ≥11 years: 180 (30.51%) | 1–5 years |
| Education | Secondary vocational education; junior college; bachelor’s degree; master’s degree or above | Junior college or below (secondary vocational education + junior college): 138 (23.39%); bachelor’s degree or above (bachelor’s degree + master’s degree or above): 452 (76.61%) | Junior college or below |
| Professional title | Nurse; senior nurse; nurse in charge; associate chief nurse or above | Nurse: 273 (46.27%); senior nurse: 161 (27.29%); nurse in charge or above (nurse in charge + associate chief nurse or above): 156 (26.44%) | Nurse |
| TCM nursing professional level | N0- N1; N2; N3- N4 | N0–N1: 261 (44.24%); N2: 178 (30.17%); N3–N4: 151 (25.59%) | N0–N1 |
| Intelligent-device use | 1–2 times/week; 3–4 times/week; 5–6 times/week; routine use | 1–2 times/week: 191 (32.37%); 3–4 times/week: 168 (28.47%); 5–6 times/week: 149 (25.25%); routine use: 82 (13.90%) | 1–2 times/week |
| Self-rated familiarity with AI | Slightly familiar; moderately familiar; fairly familiar; extremely familiar | Slightly familiar: 146 (24.75%); moderately familiar: 221 (37.46%); high familiarity (fairly familiar + extremely familiar): 223 (37.80%) | Slightly familiar |
| AI-related training | No; yes | No: 192 (32.54%); yes: 398 (67.46%) | No |

**Note.** All recategorizations were completed before R3STEP estimation on the basis of conceptual similarity and subgroup size. Percentages are calculated from N = 590. Age, technological anxiety, perceived organizational support, and general self-efficacy were entered as continuous covariates and were not recategorized. R3STEP, bias-adjusted three-step approach.
