# Supplementary material for: Latent Profiles of Attitudes Toward Artificial Intelligence Applications Among Traditional Chinese Medicine Nurses and Associated Factors: A Multicenter Cross‐Sectional Study
Source: J Nurs Manag. 2026 Nov 4;2026:6814652. doi: 10.1155/jonm/6814652 (PMC13735534; doi:10.1155/jonm/6814652)
Supplement: Supplementary file 5 [file JONM-2026-6814652-s005.docx]

**Supplementary File 7. Collinearity diagnostics for covariates included in the R3STEP model**

| **Covariate** | **VIF-equivalent** | **Tolerance** |
| --- | --- | --- |
| Age | 1.027 | 0.974 |
| Sex | 1.032 | 0.969 |
| Systematic TCM nursing training | 1.071 | 0.934 |
| TCM specialist nursing experience | 1.140 | 0.877 |
| Education | 1.028 | 0.973 |
| Professional title | 1.067 | 0.937 |
| TCM nursing professional level | 1.091 | 0.916 |
| Intelligent-device use | 1.059 | 0.944 |
| Self-rated familiarity with AI | 1.090 | 0.917 |
| AI-related training | 1.149 | 0.870 |
| Technology anxiety | 1.486 | 0.673 |
| Perceived organizational support | 2.527 | 0.396 |
| General self-efficacy | 2.483 | 0.403 |

*Note.* All 13 covariates were assessed simultaneously. VIF-equivalent values were calculated as GVIF^(1/df), and tolerance values as GVIF^(−1/df), where df is the number of parameters for each covariate. For one-degree-of-freedom terms, these are equivalent to conventional VIF and tolerance values. TCM = traditional Chinese medicine; R3STEP = bias-adjusted three-step approach.
