# Supplementary material for: Latent Profiles of Attitudes Toward Artificial Intelligence Applications Among Traditional Chinese Medicine Nurses and Associated Factors: A Multicenter Cross‐Sectional Study
Source: J Nurs Manag. 2026 Nov 4;2026:6814652. doi: 10.1155/jonm/6814652 (PMC13735534; doi:10.1155/jonm/6814652)
Supplement: Supplementary file 6 [file JONM-2026-6814652-s006.docx]

Supplementary File 8. Classification quality and sensitivity analyses

Comparison of the primary and theory-informed core-covariate R3STEP models

| **Variable/comparison** | **Profile 2 vs Profile 1** | | **Profile 3 vs Profile 1** | |
| --- | --- | --- | --- | --- |
|  | **Primary model** | Core covariate model | **Primary model** | Core covariate model |
| Age, per year | 1.005 (0.966–1.045); .818 | 1.007 (0.971–1.045); .692 | 1.015 (0.969–1.064); .528 | 1.011 (0.967–1.056); .640 |
| Technology anxiety, per point | 1.044 (1.006–1.083); .023 | 1.038 (1.004–1.073); .028 | 1.019 (0.972–1.068); .434 | 1.014 (0.971–1.059); .528 |
| Perceived organizational support, per point | 1.001 (0.968–1.036); .951 | 1.002 (0.969–1.037); .892 | 1.019 (0.981–1.058); .339 | 1.015 (0.976–1.056); .448 |
| General self-efficacy, per point | 1.008 (0.955–1.064); .780 | 1.000 (0.948–1.055); .990 | 0.948 (0.888–1.011); .103 | 0.952 (0.896–1.013); .119 |
| Male vs female | 0.985 (0.488–1.989); .967 | 0.941 (0.485–1.826); .857 | 1.019 (0.439–2.364); .965 | 0.911 (0.413–2.013); .819 |
| TCM specialist nursing experience, 6–10 vs 1–5 years | 2.461 (1.324–4.577); .004 | 2.743 (1.515–4.968); .001 | 5.331 (2.418–11.755); <.001 | 6.128 (2.890–12.991); <.001 |
| TCM specialist nursing experience, ≥11 vs 1–5 years | 2.889 (1.276–6.540); .011 | 2.865 (1.343–6.112); .006 | 15.394 (5.969–39.702); <.001 | 15.523 (6.817–35.351); <.001 |
| Intelligent-device use, 3–4 vs 1–2 times/week | 2.692 (1.469–4.934); .001 | 2.654 (1.493–4.716); .001 | 2.516 (1.129–5.607); .024 | 2.495 (1.179–5.280); .017 |
| Intelligent-device use, 5–6 vs 1–2 times/week | 1.980 (1.005–3.901); .048 | 2.099 (1.101–4.002); .024 | 2.526 (1.087–5.870); .031 | 2.986 (1.406–6.342); .004 |
| Routine intelligent-device use vs 1–2 times/week | 1.783 (0.674–4.720); .244 | 1.879 (0.744–4.740); .182 | 5.017 (1.657–15.187); .004 | 5.589 (2.054–15.205); .001 |
| Self-rated familiarity with AI: moderately familiar vs slightly familiar | 1.632 (0.932–2.859); .087 | 1.529 (0.906–2.579); .112 | 3.596 (1.501–8.612); .004 | 3.681 (1.613–8.400); .002 |
| Self-rated familiarity with AI: high familiaritya vs slightly familiar | 2.013 (1.010–4.011); .047 | 1.992 (1.031–3.849); .040 | 13.988 (5.643–34.675); <.001 | 13.842 (5.798–33.047); <.001 |
| AI-related training, yes vs no | 2.106 (1.278–3.469); .003 | 2.228 (1.379–3.599); .001 | 4.937 (2.445–9.968); <.001 | 5.329 (2.791–10.176); <.001 |

Note. Profile 1 (low AI acceptance profile) was the reference profile. The primary model simultaneously included all 13 covariates. The theory-informed core covariate model included age, sex, TCM specialist nursing experience, intelligent-device use, self-rated familiarity with AI, AI-related training, technology anxiety, perceived organizational support, and general self-efficacy; systematic TCM nursing training, education, professional title, and TCM nursing professional level were omitted. Values are adjusted odds ratios (95% confidence intervals); P values are from R3STEP logit-coefficient tests. aOR, adjusted odds ratio; CI, confidence interval. a High familiarity combined the fairly familiar and extremely familiar categories.

**Average posterior probability matrix for the primary three-profile model**

| **Most-likely profile membership** | **Profile 1** | **Profile 2** | **Profile 3** |
| --- | --- | --- | --- |
| Profile 1 | .967 | .033 | .000 |
| Profile 2 | .010 | .972 | .019 |
| Profile 3 | .000 | .023 | .977 |

**Targeted local-independence sensitivity analysis**

| **Characteristic** | **Primary model** | **Sensitivity model** |
| --- | --- | --- |
| Within-profile residual covariances | Fixed at zero | Q1-Q4, Q2-Q6, and Q7-Q15 freely estimated and constrained equal across profiles |
| Residual covariance estimates | Not applicable | .141, .131, and .124, respectively; all P < .001 |
| Estimated profile proportions | 25.14%, 39.68%, and 35.18% | 24.26%, 39.33%, and 36.41% |
| Entropy | .939 | .934 |
| Diagonal average posterior probabilities | .967, .972, and .977 | .970, .964, and .972 |
| Agreement with primary modal classification | Reference | 97.46% (575/590) |
| Adjusted Rand index | Reference | .923 |

**Alternative indicator specifications**

| **Alternative indicators** | **Estimated profile proportions (low/moderate/high)** | **Entropy** | **Result** |
| --- | --- | --- | --- |
| Mean scores of the two prespecified domains | 20.24% / 41.04% / 38.73% | .862 | Reproduced the ordered low-, moderate-, and high-attitude pattern |
| CFA-derived regression factor scores | 21.05% / 40.50% / 38.46% | .880 | Reproduced the ordered low-, moderate-, and high-attitude pattern |
