# Supplementary material for: Latent Profiles of Attitudes Toward Artificial Intelligence Applications Among Traditional Chinese Medicine Nurses and Associated Factors: A Multicenter Cross‐Sectional Study
Source: J Nurs Manag. 2026 Nov 4;2026:6814652. doi: 10.1155/jonm/6814652 (PMC13735534; doi:10.1155/jonm/6814652)
Supplement: Supplementary file 7 [file JONM-2026-6814652-s007.docx]

**STROBE Statement—Checklist of items that should be included in reports of cross-sectional studies**

|  | Item No | Recommendation | **Page / No.** |
| --- | --- | --- | --- |
| Title and abstract | 1 | (*a*) Indicate the study’s design with a commonly used term in the title or the abstract | p. 1. The title and Abstract identify the study as a multicenter cross-sectional study or survey. |
|  |  | (*b*) Provide in the abstract an informative and balanced summary of what was done and what was found | pp. 1–2. The Abstract summarizes the background, survey setting and dates, sample size, item-level LPA, R3STEP adjustment for 13 covariates, three profiles, associated factors, and conclusions. |
| Introduction | | | |
| Background/rationale | 2 | Explain the scientific background and rationale for the investigation being reported | pp. 2–5. The Introduction describes AI in nursing, the TCM nursing context, TAM, existing evidence, the research gap, and the rationale for LPA. |
| Objectives | 3 | State specific objectives, including any prespecified hypotheses | p. 5. The final Introduction paragraph states the objectives: to identify profiles of attitudes toward AI applications among TCM nurses and examine factors associated with profile membership. |
| Methods | | | |
| Study design | 4 | Present key elements of study design early in the paper | p. 5. Study design describes the multicenter cross-sectional survey, questionnaire measures, item-level LPA, and analysis of associated factors. |
| Setting | 5 | Describe the setting, locations, and relevant dates, including periods of recruitment, exposure, follow-up, and data collection | pp. 5–6 and 9–10. Participants identifies three hospitals and 30 affiliated community health service centers in South China. Data collection and quality control reports the online survey period, 10 January to 7 April 2026. |
| Participants | 6 | (*a*) Give the eligibility criteria, and the sources and methods of selection of participants | pp. 5–6. Participants describes the participating institutions, multicenter convenience sampling, inclusion criteria, and exclusion criteria. |
| Variables | 7 | Clearly define all outcomes, exposures, predictors, potential confounders, and effect modifiers. Give diagnostic criteria, if applicable | pp. 5 and 7–12; Tables 1 and 5 (pp. 35–36 and 42). Study design, Measures, and Statistical analysis describe attitude-profile membership, demographic and occupational characteristics, AI-related experience, technology anxiety, perceived organizational support, and general self-efficacy. Coding and reference categories are cited in Supplementary Files 2 and 6. |
| Data sources/ measurement | 8* | For each variable of interest, give sources of data and details of methods of assessment (measurement). Describe comparability of assessment methods if there is more than one group | pp. 7–9 and 14. Measures describes the questionnaire, instrument sources, Chinese adaptation, response formats, and scoring. Results reports sample-specific psychometric findings. Full administered attitude and technology-anxiety items are cited in Supplementary File 5. |
| Bias | 9 | Describe any efforts to address potential sources of bias | pp. 9–10, 12–13, and 23–24. Data collection and quality control and Quality Control Measures describe anonymous voluntary responses, one submission per WeChat account, mandatory items, and questionnaire screening. Limitations discusses convenience sampling and self-assessment and measurement limitations. |
| Study size | 10 | Explain how the study size was arrived at | pp. 6–7. Sample size reports the single-proportion calculation, the 20% allowance for invalid questionnaires, the target of 481, the final sample of 590, and LPA-specific adequacy considerations. |
| Quantitative variables | 11 | Explain how quantitative variables were handled in the analyses. If applicable, describe which groupings were chosen and why | pp. 7–12; Tables 1, 4, and 5 (pp. 35–36 and 40–42). Measures and Statistical analysis describe scale scoring, continuous-variable summaries and comparisons, categorical coding, reference groups, and the four-category treatment of intelligent-device use. Coding details are cited in Supplementary Files 2 and 6. |
| Statistical methods | 12 | (*a*) Describe all statistical methods, including those used to control for confounding | pp. 10–12. Statistical analysis describes normality assessment, descriptive comparisons, reliability and internal-validation CFA, LPA parameterization and enumeration, and bias-adjusted R3STEP with simultaneous adjustment for 13 a priori covariates. |
|  |  | (*b*) Describe any methods used to examine subgroups and interactions | Not applicable to the analyses reported in this manuscript. |
|  |  | (*c*) Explain how missing data were addressed | pp. 9 and 12–13. All questionnaire items were mandatory. Quality Control Measures states that the final analytic sample had no item-level missing data and that neither imputation nor full-information maximum likelihood was required. |
|  |  | (*d*) If applicable, describe analytical methods taking account of sampling strategy | pp. 5–6. Participants reports multicenter convenience sampling. |
|  |  | (*e*) Describe any sensitivity analyses | pp. 11–12 and 15–17. Statistical analysis and Results describe targeted local-independence sensitivity analysis, alternative indicator specifications, and a nine-covariate R3STEP sensitivity model. Details are cited in Supplementary File 8. |
| Results | | | |
| Participants | 13* | (a) Report numbers of individuals at each stage of study—eg numbers potentially eligible, examined for eligibility, confirmed eligible, included in the study, completing follow-up, and analysed | p. 13; Figure 1 (p. 34). Results reports 654 returned questionnaires, 64 exclusions, and 590 included in the final analysis. Figure 1 gives returned and included counts for the hospitals and the community health service network. |

|  | Item No | Recommendation | Page / No. |
| --- | --- | --- | --- |
|  |  | (b) Give reasons for non-participation at each stage | pp. 12–13; Figure 1 (p. 34). Quality Control Measures and Results describe questionnaire exclusion criteria. Results reports 31 exclusions for completion time under 100 seconds, 14 for logical inconsistency, and 19 for patterned responding. |
|  |  | (c) Consider use of a flow diagram | Figure 1 (p. 34) presents questionnaire return, screening, exclusion, and inclusion in the final analysis. |
| Descriptive data | 14* | (a) Give characteristics of study participants (eg demographic, clinical, social) and information on exposures and potential confounders | p. 13; Table 1 (pp. 35–36) and Table 4 (pp. 40–41). Results and the tables report participant demographic, occupational, and AI-related characteristics and profile-specific comparisons. |
|  |  | (b) Indicate number of participants with missing data for each variable of interest | pp. 12–13. Quality Control Measures states that all questionnaire items were mandatory and that no item-level missing data were present in the final analytic sample. |
| Outcome data | 15* | Report numbers of outcome events or summary measures | pp. 13–16; Table 2 (p. 37), Figure 2 (p. 38), and Table 3 (p. 39). Results reports summary scores, psychometric findings, model-fit indices, profile proportions, and classification quality. Figure 2 presents model-estimated item means; Table 3 presents observed item means and standard deviations by most-likely profile assignment. |
| Main results | 16 | (*a*) Give unadjusted estimates and, if applicable, confounder-adjusted estimates and their precision (eg, 95% confidence interval). Make clear which confounders were adjusted for and why they were included | pp. 11–12 and 16–17; Table 4 (pp. 40–41) and Table 5 (p. 42). Table 4 reports profile-specific descriptive comparisons. Table 5 reports adjusted odds ratios, 95% confidence intervals, and P values from R3STEP. Statistical analysis describes simultaneous adjustment for 13 a priori covariates and their selection basis. |
|  |  | (*b*) Report category boundaries when continuous variables were categorized | pp. 7–12; Table 1 (pp. 35–36) and Table 5 (p. 42). Measures and Statistical analysis describe scale ranges, analytic categories, and reference groups. Tables report category boundaries; detailed coding and recategorization are cited in Supplementary Files 2 and 6. |
|  |  | (*c*) If relevant, consider translating estimates of relative risk into absolute risk for a meaningful time period | Not applicable to this cross-sectional study. |
| Other analyses | 17 | Report other analyses done—eg analyses of subgroups and interactions, and sensitivity analyses | pp. 11–12 and 15–17. Statistical analysis and Results describe local-independence sensitivity analysis, alternative indicator specifications, and the core-covariate R3STEP sensitivity model. Sensitivity results and collinearity diagnostics are cited in Supplementary Files 8 and 7, respectively. |
| Discussion | | | |
| Key results | 18 | Summarise key results with reference to study objectives | p. 17. Summary of main findings summarizes the overall attitude score, the three profiles, and factors associated with profile membership. |
| Limitations | 19 | Discuss limitations of the study, taking into account sources of potential bias or imprecision. Discuss both direction and magnitude of any potential bias | pp. 23–24. Limitations and future research discusses the cross-sectional design and unclear temporal ordering, possible reciprocal associations, convenience sampling and limited generalizability, use of general organizational support and self-efficacy measures, device-use and AI-familiarity response-option limitations, possible misclassification, and self-assessment bias. |
| Interpretation | 20 | Give a cautious overall interpretation of results considering objectives, limitations, multiplicity of analyses, results from similar studies, and other relevant evidence | pp. 17–25. Discussion and Conclusion interpret profile patterns and associated factors in relation to the study objectives, previous research, TAM, study limitations, nursing implications, and future research. |
| Generalisability | 21 | Discuss the generalisability (external validity) of the study results | pp. 23–24. Limitations and future research notes convenience sampling in South China and the need to validate generalizability in other regions and hospitals of different levels. |
| Other information | | | |
| Funding | 22 | Give the source of funding and the role of the funders for the present study and, if applicable, for the original study on which the present article is based | p. 26. Funding identifies the Shenzhen Key TCM Specialty Construction Project (No. ZYTS019) and states that the funder had no role in study design, data collection, data analysis, manuscript preparation, or the decision to submit the manuscript for publication. |

**Note:** An Explanation and Elaboration article discusses each checklist item and gives methodological background and published examples of transparent reporting. The STROBE checklist is best used in conjunction with this article (freely available on the Web sites of PLoS Medicine at http://www.plosmedicine.org/, Annals of Internal Medicine at http://www.annals.org/, and Epidemiology at http://www.epidem.com/). Information on the STROBE Initiative is available at www.strobe-statement.org.
