# Supplementary material for: Latent Profiles of Attitudes Toward Artificial Intelligence Applications Among Traditional Chinese Medicine Nurses and Associated Factors: A Multicenter Cross‐Sectional Study
Source: J Nurs Manag. 2026 Nov 4;2026:6814652. doi: 10.1155/jonm/6814652 (PMC13735534; doi:10.1155/jonm/6814652)
Supplement: Supplementary file 8 [file JONM-2026-6814652-s008.docx]

**Supplementary File 1. General Information Questionnaire**

Latent profiles of attitudes toward artificial intelligence applications among traditional Chinese medicine nurses and associated factors

**Instructions:** This questionnaire was used to collect sociodemographic characteristics, occupational characteristics, and AI-related experience among traditional Chinese medicine (TCM) nurses. Please select the option that best describes your actual situation unless otherwise specified.

**1. Age**

Please enter your actual age: ____ years.

**2. Gender**

□ Female

□ Male

**3. Systematic TCM nursing training**

□ No

□ Yes

**4. Years of TCM specialist nursing experience**

□ 1-5 years

□ 6-10 years

□ 11 years or more

**5. Education**

□ Secondary vocational education

□ Junior college

□ Bachelor

□ Master’s degree and above

**6. Professional title**

□ Nurse

□ Senior nurse

□ Nurse in charge

□ Associate chief nurse and above

**7. TCM nursing professional level**

□ N0-N1 (Junior)

□ N2 (Intermediate)

□ N3-N4 (Senior)

**8. Frequency of using intelligent devices in TCM nursing**

□ 1-2 times per week

□ 3-4 times per week

□ 5-6 times per week

□ Regularly used as routine practice

**9. Self-rated familiarity with AI**

□ Slightly familiar

□ Moderately familiar

□ Fairly familiar

□ Extremely familiar

**10. AI-related training experience**

□ No

□ Yes
