# Supplementary material for: Latent Profiles of Attitudes Toward Artificial Intelligence Applications Among Traditional Chinese Medicine Nurses and Associated Factors: A Multicenter Cross‐Sectional Study
Source: J Nurs Manag. 2026 Nov 4;2026:6814652. doi: 10.1155/jonm/6814652 (PMC13735534; doi:10.1155/jonm/6814652)
Supplement: Supplementary file 9 [file JONM-2026-6814652-s009.docx]

**Supplementary File 5**

# S1. Attitude Scale towards the Use of Artificial Intelligence Technologies in Nursing (ASUAITIN-C)

**Source and cultural adaptation.** The original 15-item Attitude Scale towards the Use of Artificial Intelligence Technologies in Nursing (ASUAITIN) was developed by Yılmaz et al. [1]. Wang et al. [2] translated and culturally adapted the scale for Chinese nurses through independent forward translation, reconciliation, independent back-translation, comparison with the source version, review by the original developer, expert consultation, and pilot testing in 30 nurses. Items 3, 10, 11, and 13 were linguistically adapted for the Chinese nursing context. The Chinese version retains the two-domain organization of the original instrument: negative attitudes (items 1–6) and positive attitudes (items 7–15).

**Response format and scoring.** Each item is rated from 1 = strongly disagree to 5 = strongly agree. Items 1–6 are reverse-scored as 6 minus the original response, whereas items 7–15 are scored directly. The 15 scored item responses are summed to yield a possible total score of 15–75, with higher scores indicating a more favorable attitude toward AI applications in nursing. The instrument was administered in Chinese; English translations of the administered items are provided below.

**Table S1. Full item set and scoring of the ASUAITIN-C**

| **Code** | **Original domain** | **English translation of administered Chinese item** | **Scoring** |
| --- | --- | --- | --- |
| Q1 | Negative attitude | I think artificial intelligence technology may hinder the advancement of nursing practice.  我认为人工智能技术将成为护理实践应用的阻碍 | Reverse (6 − response) |
| Q2 | Negative attitude | I feel uncomfortable when I think about how artificial intelligence technologies will be used in nursing care in the future.  当我想到人工智能技术在未来护理工作中的应用,我感到不适 | Reverse (6 − response) |
| Q3 | Negative attitude | I think that the nursing profession will be harmed if artificial intelligence technologies are used more in the future.  我认为如果未来人工智能技术被广泛使用,护理行业将遭受不利影响 | Reverse (6 − response) |
| Q4 | Negative attitude | I think that the use of artificial intelligence technologies in nursing care will put patient safety at risk.  我认为在护理工作中使用人工智能技术会让患者安全遭受风险 | Reverse (6 − response) |
| Q5 | Negative attitude | I think it is not right to use artificial intelligence technologies in nursing.  我认为在护理领域中使用人工智能技术是不对的 | Reverse (6 − response) |
| Q6 | Negative attitude | I think that the use of artificial intelligence technologies in nursing practice may cause ethical problems.  我认为在护理实践中使用人工智能技术会引发伦理问题 | Reverse (6 − response) |
| Q7 | Positive attitude | Artificial intelligence technologies can provide new opportunities for nurses.  人工智能技术能够为护士提供新的机遇 | Direct (1–5) |
| Q8 | Positive attitude | The field of use of artificial intelligence technologies in nursing is wide.  人工智能技术在护理领域中的使用范围广泛 | Direct (1–5) |
| Q9 | Positive attitude | There are many beneficial applications of artificial intelligence technologies in nursing.  人工智能技术在护理领域中有诸多有益的应用 | Direct (1–5) |
| Q10 | Positive attitude | I would prefer to use a system with artificial intelligence for some routine nursing procedures, such as vital-sign and pain assessment.  对于一些常规的护理操作(如生命体征监测和疼痛评估),我更倾向于使用有人工智能辅助的系统 | Direct (1–5) |
| Q11 | Positive attitude | Predictions about a patient generated by artificial intelligence technologies (e.g., nursing diagnosis and care needs) can provide practical opportunities and make nursing work easier.  人工智能技术对患者的预测(如护理诊断、护理需求等)能够提供实际的机会,使护理职业更轻松 | Direct (1–5) |
| Q12 | Positive attitude | I think that benefiting from artificial intelligence technologies in nursing is achievable.  我认为,护理领域从人工智能技术中获益是可实现的 | Direct (1–5) |
| Q13 | Positive attitude | I would like to acquire skills in learning and using artificial intelligence technologies in nursing.  我希望具备在护理实践中学习和运用人工智能技术的能力 | Direct (1–5) |
| Q14 | Positive attitude | I would like to receive training on the use of artificial intelligence technologies in nursing.  我希望接受关于在护理实践中学习和使用人工智能技术的培训 | Direct (1–5) |
| Q15 | Positive attitude | I think it is necessary to include artificial intelligence technologies in the core curriculum of nursing education.  我认为有必要将人工智能技术纳入护理教育的核心课程 | Direct (1–5) |

# Note：All items were rated on a 5-point Likert scale ranging from 1 (strongly disagree) to 5 (strongly agree). Items Q1–Q6 were reverse-scored using the formula “6 − original response,” whereas items Q7–Q15 were scored directly. The total score was calculated by summing the recoded scores of Q1–Q6 and the original scores of Q7–Q15, yielding a possible range of 15–75. Higher total scores indicate a more favorable attitude toward the use of artificial intelligence technologies in nursing. The Chinese wording presented in the table is the version administered to participants; the English wording is provided as a translation for international readers.

# S2. Chinese version of the Technology Anxiety Scale

**Source and cultural adaptation.** The Technophobia Scale was developed by Khasawneh [3]. Sun et al. [4] translated, back-translated, and culturally adapted the instrument for Chinese older adults, producing a 13-item Chinese Technology Anxiety Scale with three dimensions: technology-related tension (items 1–5), fear of technology (items 6–10), and privacy and security concerns (items 11–13). In the present survey, the 13 Chinese items were administered using a five-category response format.

**Response format and scoring.** Each item was rated on a 5-point Likert scale ranging from 1 (strongly disagree) to 5 (strongly agree). No item was reverse-scored. The 13 item scores were summed to yield a possible total score of 13–65, with higher scores indicating greater technology anxiety.

**Table S2. Full item set and scoring of the Chinese Technology Anxiety Scale**

| **Analysis code** | **Original item No.** | **Dimension** | **English translation of administered Chinese item** | **Score** |  |
| --- | --- | --- | --- | --- | --- |
| TT1 | 11 | Technology-related tension | I feel uneasy when I have to use a new communication device (e.g., a new mobile phone or tablet computer).  当我必须使用新的通讯设备（新手机、新掌上电脑）时，我会感到不安 | Direct (1–5) |  |
| TT2 | 16 | Technology-related tension | I avoid replacing communication devices (e.g., changing my mobile phone) whenever possible because doing so makes me nervous.  我尽可能地避免更换通讯设备（如换手机），因为这使我感到紧张 | Direct (1–5) |  |
| TT3 | 15 | Technology-related tension | I avoid using new technologies, such as smartphones and smart wristbands, whenever possible.  我尽可能地避免使用新的技术，如智能手机、智能手环等 | Direct (1–5) |  |
| TT4 | 12 | Technology-related tension | I feel uneasy when I have to learn a new operating system (e.g., updating a mobile-phone system or upgrading a computer operating system).  当我必须学习新的操作系统时（如更新手机系统、更新电脑windows7到8）时，我 会感到不安 | Direct (1–5) |  |
| TT5 | 9 | Technology-related tension | I do not dare to use online search engines such as Baidu, 360 Search, or Sogou.  我不敢使用百度、360搜索、搜狗等网络搜索引擎 | Direct (1–5) |  |
| TF1 | 4 | Fear of technology | I am afraid that new technologies will replace my job in the future.  我害怕新技术未来会取代我的工作 | Direct (1–5) |  |
| TF2 | 3 | Fear of technology | I am afraid that, sooner or later, new technologies will make humans obsolete.  我害怕迟早有一天新技术会让我们（人类）被淘汰 | Direct (1–5) |  |
| TF3 | 7 | Fear of technology | I worry about new technologies because they may affect my life emotionally, physically, and psychologically.  我担心新技术，是因为技术会在情感上、生理上和心理上影响我的生活 | Direct (1–5) |  |
| TF4 | 2 | Fear of technology | I am very afraid that technology will change how we live, communicate, love, and even judge other people.  我非常害怕技术会改变我们的生活、沟通、爱、甚至评判他人的方式 | Direct (1–5) |  |
| TF5 | 6 | Fear of technology | I worry about new technologies because, if technology fails, I may be unable to function and feel as though I have returned to the Stone Age.  我担心新技术，是因为一旦技术出现故障（如某些原因导致技术失灵），我会回到 石器时代而寸步难行 | Direct (1–5) |  |
| PS1 | 1 | Privacy and security concerns | I am afraid that someone may use technology to listen to or monitor everything I do.  我害怕有人用技术时刻监听、监视我的所作所为 | Direct (1–5) |  |
| PS2 | 10 | Privacy and security concerns | I am very afraid that traces left online after connecting to the Internet may be tracked by others.  我非常害怕连接互联网后，在网络上留下痕迹可能会被人追踪 | Direct (1–5) |  |
| PS3 | 14 | Privacy and security concerns | I am afraid of search websites such as Baidu, 360 Search, and Sogou because they may make it easier for others to track or monitor me.  我害怕百度、360搜索、搜狗等搜索网站，是担心它们更容易让别人跟踪监控我 | Direct (1–5) |  |

# S3. Psychometric properties in the present sample

Internal consistency was evaluated using Cronbach’s *α* for each total scale and subscale. For the attitude scale, a post hoc random split-sample fixed-parameter internal-validation CFA was conducted to evaluate a general factor shared by the 15 scored items [5]. Using a fixed random seed (20260826), participants were divided equally into a derivation subset (n = 295) and a validation subset (n = 295). Because the attitude items had five response categories, the scored responses were treated as approximately continuous. A one-factor model was estimated in the derivation subset using robust maximum likelihood (MLR). The 15 unstandardized factor loadings, 15 item residual variances, and 15 item intercepts estimated in the derivation subset were fixed before evaluation in the validation subset; only the latent mean and variance were freely estimated in validation. Robust χ², CFI, TLI, RMSEA with its 90% confidence interval, and SRMR were reported [6,7]. Composite reliability (CR) and average variance extracted (AVE) were calculated from the derivation-subset estimates [8].

The three-factor structure of the Technology Anxiety Scale was evaluated in the full sample using weighted least squares mean- and variance-adjusted estimation (WLSMV), theta parameterization, and all 13 five-category items specified as ordered indicators. Scaled χ², robust CFI and TLI, robust RMSEA with its 90% confidence interval, SRMR, standardized factor loadings, and factor correlations were reported. Psychometric analyses were performed in R version 4.2.3 using lavaan version 0.6-19 [6].

| **Table S3. Descriptive statistics and internal consistency of the two core instruments (N = 590)** | | | | | |
| --- | --- | --- | --- | --- | --- |
| Instrument/dimension | Items | M (SD) | Observed range | Possible range | Cronbach’s *α* |
| ASUAITIN-C total | 15 | 48.13 (13.49) | 16–75 | 15–75 | 0.971 |
| Negative-attitude items, reverse-scored | 6 | 18.29 (5.57) | 6–30 | 6–30 | 0.930 |
| Positive-attitude items | 9 | 29.83 (8.11) | 10–45 | 9–45 | 0.951 |
| Technology Anxiety Scale total | 13 | 23.94 (8.35) | 13–65 | 13–65 | 0.936 |
| Technology-related tension | 5 | 9.23 (3.41) | 5–25 | 5–25 | 0.851 |
| Fear of technology | 5 | 9.04 (3.37) | 5–25 | 5–25 | 0.869 |
| Privacy and security concerns | 3 | 5.67 (2.29) | 3–15 | 3–15 | 0.827 |

**Table S4. Descriptive statistics and internal consistency of the two core instruments (N = 590)**

| **Instrument** | **Scale / dimension** | **Items** | **Cronbach’s *α*** |
| --- | --- | --- | --- |
| ASUAITIN-C | Total scale | 15 | 0.971 |
| ASUAITIN-C | Negative-attitude items (reverse-scored) | 6 | 0.930 |
| ASUAITIN-C | Positive-attitude items | 9 | 0.951 |
| Technology Anxiety Scale | Total scale | 13 | 0.936 |
| Technology Anxiety Scale | Technology-related tension | 5 | 0.851 |
| Technology Anxiety Scale | Fear of technology | 5 | 0.869 |
| Technology Anxiety Scale | Privacy and security concerns | 3 | 0.827 |
| Perceived Organizational Support Scale | Total scale | 13 | 0.950 |
| General Self-Efficacy Scale | Total scale | 10 | 0.927 |

| **Table S5. Split-sample fixed-parameter internal-validation CFA of the ASUAITIN-C** | | |
| --- | --- | --- |
| **Model** | **Estimator** | **Derivation-sample estimates** |
| One-factor fixed-parameter model | MLR | Standardized loadings = .817–.855; CR = .972; AVE = .701 |

Note. Participants were randomly divided into derivation and validation subsets (n = 295 each; seed = 20260826). Fifteen factor loadings, 15 item residual variances, and 15 item intercepts estimated in the derivation subset were fixed in the validation subset; only the latent mean and variance were freely estimated. The five-category scored items were treated as approximately continuous. Factor loadings, CR, and AVE are from the derivation subset; fit indices are from the validation subset.

| **Table S6. Three-factor ordinal CFA of the Technology Anxiety Scale (N = 590)** | | | | |
| --- | --- | --- | --- | --- |
| **Model** | **Estimator** | **Fit indices** | **Standardized loadings** | **Factor correlations** |
| Three-factor model | WLSMV | scaled χ²(62) = 155.712; p < .001; robust CFI = .968; robust TLI = .960; robust RMSEA = .066 (90% CI [.053, .080]); SRMR = .032 | .704–.839 | .819–.936 |

**Note.** All 13 five-category items were treated as ordered categorical indicators. WLSMV estimation with theta parameterization was used. The χ² value is the scaled statistic; CFI, TLI, and RMSEA are the robust indices.

# References

[1] Yılmaz D., Uzelli D., and Dikmen Y., Psychometrics of the Attitude Scale Towards the Use of Artificial Intelligence Technologies in Nursing, BMC Nursing. (2025) 24, no. 1, 151, <https://doi.org/10.1186/s12912-025-02732-7>
[2] Wang Y. Y., Xue M. X., Min Y., et al., Chinese Translation and Psychometric Validation of the Attitude Scale Towards the Use of Artificial Intelligence Technologies in Nursing [in Chinese], Journal of Nursing Science. (2026) 41, no. 2, 12–15, 25.
[3] Khasawneh O. Y., Technophobia: Examining Its Hidden Factors and Defining It, Technology in Society. (2018) 54, 93–100, <https://doi.org/10.1016/j.techsoc.2018.03.008> .
[4] Sun E. H., Gao Y., and Ye X. C., Chinese Translation of the Technology Anxiety Scale and Its Reliability and Validity Among Older Adults [in Chinese], Chinese Journal of Nursing. (2022) 57, no. 3, 380–384, <https://doi.org/10.3761/j.issn.0254-1769.2022.03.021> .
[5] Cudeck R. and Browne M. W., Cross-Validation of Covariance Structures, Multivariate Behavioral Research. (1983) 18, no. 2, 147–167, <https://doi.org/10.1207/s15327906mbr1802_2> .
[6] Rosseel Y., lavaan: An R Package for Structural Equation Modeling, Journal of Statistical Software. (2012) 48, no. 2, 1–36, <https://doi.org/10.18637/jss.v048.i02> .
[7] Hu, L., & Bentler, P. M. (1999). Cutoff criteria for fit indexes in covariance structure analysis: Conventional criteria versus new alternatives. Structural Equation Modeling: A Multidisciplinary Journal, 6(1), 1–55. <https://doi.org/10.1080/10705519909540118>
[8] Fornell C. and Larcker D. F., Evaluating Structural Equation Models With Unobservable Variables and Measurement Error, Journal of Marketing Research. (1981) 18, no. 1, 39–50, <https://doi.org/10.1177/002224378101800104>.
